# Triethyl Phosphate Enables Regulated Solvation Structure and Robust Anode Interphase in Dual-Salt Mg(OTf)_2_‑MgCl_2_ Electrolytes for Rechargeable Magnesium Batteries

**DOI:** 10.1021/acscentsci.6c01164

**Published:** 2026-09-07

**Authors:** Yan Liu, Li Qian, Xiaogang Li, Yuqing Tian, Jiaping Ma, Siyu Song, Yixian Zhang, Aihua Yuan, Huajun Tian

**Affiliations:** † School of Environmental and Chemical Engineering, 118308 Jiangsu University of Science and Technology, Zhenjiang 212100, PR China; ‡ Beijing Laboratory of New Energy Storage Technology and Key Laboratory of Power Station Energy Transfer Conversion and System of Ministry of Education, School of Energy Power and Mechanical Engineering, North China Electric Power University, Beijing 102206, PR China

## Abstract

Rechargeable magnesium batteries are promising next-generation energy storage systems owing to the low cost and high natural abundance of magnesium. However, their practical application is critically impeded by the parasitic decomposition of conventional electrolytes at the Mg anode, leading to the formation of a passivation layer, high desolvation barriers, and poor cycling stability. Herein, triethyl phosphate (TEP) is introduced as a cosolvent into the Mg­(OTf)_2_ + MgCl_2_-DME electrolyte to regulate solvation structures and interfacial chemistry. Comprehensive spectroscopic and electrochemical analyses reveal that TEP facilitates salt dissociation and reconstructs the Mg^2+^ solvation structure, thereby reducing the desolvation barrier and promoting ion transport. Meanwhile, TEP contributes to the formation of a stable and robust solid electrolyte interphase on the Mg anode, effectively suppressing side reactions and enabling uniform Mg deposition. The Mg||Mg symmetric cell exhibits stable cycling for over 3500 h with an overpotential below 0.2 V at 0.1 mA cm^–2^ and 0.05 mAh cm^–2^. The Mg||Mo asymmetric cell delivers a Coulombic efficiency of 99.4% after 3000 h at 0.5 mA cm^–2^ and 0.25 mAh cm^–2^. Furthermore, the Mg||α-MnO_2_ full cell maintains a discharge capacity of 176.9 mAh g^–1^ after 350 cycles at 0.1 C. This work provides a solvation-regulation strategy for designing high-performance Mg electrolytes.

## Graphical abstract

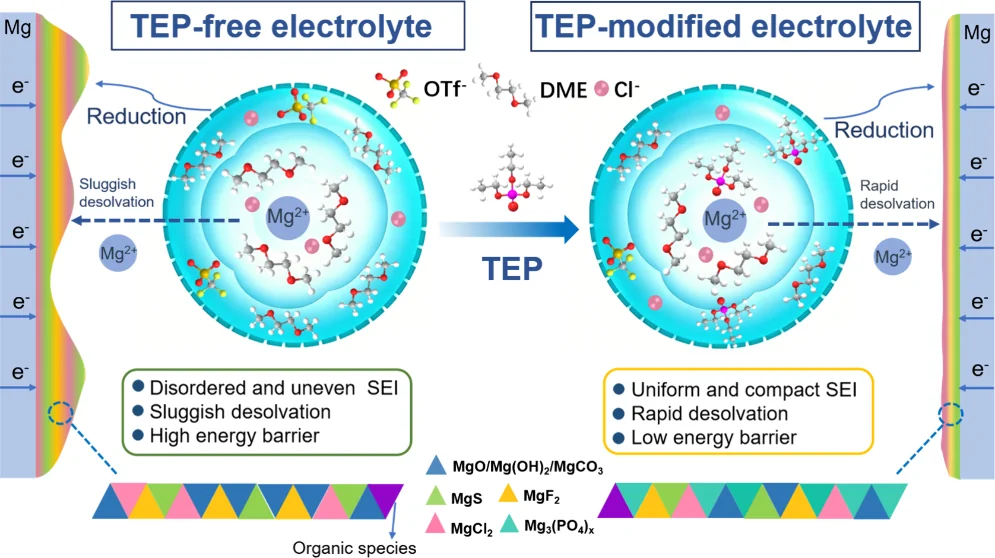

## 1 Introduction

Rechargeable magnesium batteries (RMBs) become a highly promising candidate to lithium-ion batteries for the large-scale energy storage system due to a high volumetric specific capacity (3833 mAh cm^–3^), a low reduction potential (−2.37 V vs SHE), abundant reserves (≈23300 ppm in the earth’s crust), and good air-stability of Mg metal anode.^1–3^ In addition, the deposition/dissolution processes of Mg are usually uniform in most as-reported electrolytes, and the deposited Mg is less likely to form dendrites on the anode, which enables high safety for RMBs.^4–6^ Unfortunately, the traditional electrolytes, such as Mg­(PF_6_)_2_, magnesium bis­(trifluoromethanesulfonimide) (Mg­(TFSI)_2_) and Mg­(ClO_4_)_2_ in ether or ester solvents, generally react with Mg anode, forming passivation layers that block the Mg^2+^ transportation and leads to higher deposition/dissolution overpotential.^7,8^ Therefore, developing novel electrolytes that are both Mg^2+^-conductive and capable of achieving reversible Mg plating/stripping is vital for the technical viability of RMBs.^9–11^

Among numerous candidate electrolyte systems, Magnesium trifluoromethanesulfonate, also known as magnesium triflate (Mg­(OTf)_2_), has attracted extensive attention due to its non-nucleophilicity, low cost, and easy handling. Compared with Mg­(TFSI)_2_, the anion of Mg­(OTf)_2_ has weaker coordination ability, which is theoretically more conducive to the desolvation of Mg^2+^, thereby enhancing the deposition/dissolution kinetics. However, Mg­(OTf)_2_ suffers from poor solubility in a single ether solvent such as 1,2-dimethoxyethane (DME) and tetrahydrofuran­(THF), and the Mg­(OTf)_2_/ether electrolytes also passivate the Mg metal anode, resulting in low CE, which severely limits its application.^12,13^ To address the above challenges, researchers have proposed a ″dual-salt strategy″, i.e., introducing a second magnesium salt to synergistically regulate the solvation structure and interfacial chemistry of the electrolyte. Among them, magnesium chloride (MgCl_2_), as a classic Lewis acid additive, has been proven to effectively improve the performance of Mg­(OTf)_2_-based electrolytes. On the one hand, the addition of MgCl_2_ can significantly increase the solubility of Mg­(OTf)_2_ in ether solvents, promote salt dissociation, and increase carrier concentration. On the other hand, Cl^–^ ions can be preferentially adsorbed on the surface of the magnesium metal anode, inhibiting the reductive decomposition of impurity water and OTf^–^ anions through dynamic competition, thereby suppressing the formation of interfacial passivation films and optimizing the Mg^2+^ deposition/dissolution process.^14^ Although the synergistic effect of MgCl_2_ has been widely verified, the Mg­(OTf)_2_+MgCl_2_-DME binary electrolyte still faces severe inherent challenges. The migration and deposition behavior of Mg^2+^ is limited to partial regions on the magnesium anode surface, which easily triggers uneven electric field distribution and irregular magnesium deposition morphology, ultimately impairing the reversibility of the Mg electrode process. Furthermore, severe Mg anode passivation derived from the strong adsorption of OTf^–^ anions and solvated DME molecules cannot be effectively eliminated, further deteriorating interfacial reaction kinetics and long-term cycling performance.

In recent advances of divalent metal battery electrolytes, introducing functional additives has been widely recognized as a highly efficient and universal strategy to tailor cationic solvation coordination environments. This approach fundamentally reshapes interfacial reaction pathways, suppresses detrimental parasitic side reactions, and elevates the redox reversibility and kinetic dynamics of multivalent metal deposition/dissolution processes. By reconstructing the solvation sheath of divalent cations, rationally engineered additives can optimize ion-pair dissociation behaviors, reduce interfacial Mg^2+^ desolvation energy barriers, and direct the in situ growth of robust, ion-permeable SEI.^15–19^ These merits collectively alleviate long-standing bottlenecks in conventional divalent battery systems, including sluggish interfacial kinetics, aggravated anode corrosion, and fragile electrode–electrolyte interfaces.

Phosphate-based compounds have shown great potential in the design of advanced magnesium battery electrolytes due to the unique coordination properties and interfacial regulation capabilities.^20,21^ As a typical representative, TEP not only possesses good flame retardancy to improve the thermal stability of the electrolyte but also can precisely regulate the solvation sheath of Mg^2+^ through the steric hindrance effect. Studies have shown that TEP molecules can moderately replace ether solvent molecules in the coordination shell of Mg^2+^, weaken the interaction between Mg^2+^ and solvents/anions, effectively reduce the Mg^2+^ desolvation energy barrier, and accelerate interfacial charge transfer.^22,23^ Meanwhile, the preferential reduction of TEP on the anode surface can in situ form an inorganic SEI rich in Mg­(PO_4_)_*x*_. This interfacial layer shows both ionic conductivity and electronic insulation, which can not only inhibit the continuous decomposition of the electrolyte, but also guide the uniform Mg deposition, significantly improving the cycle stability of batteries.^24^ Nevertheless, previous studies involving TEP have mainly focused on single-salt electrolyte systems or nonmagnesium battery chemistries, where TEP was generally employed as a bulk solvent component or a conventional additive to improve electrolyte stability, safety, or interfacial compatibility. In these cases, the role of TEP was primarily associated with modifying the overall physicochemical properties of the electrolyte, rather than precisely regulating Mg^2+^ solvation chemistry and interfacial reactions. In contrast, our work focuses on an OTf^–^/Cl^–^ dual-salt Mg­(OTf)_2_+MgCl_2_-DME electrolyte, in which TEP is introduced as a functional cosolvent rather than a simple additive. Unlike conventional additive strategies, TEP in our electrolyte participates in the Mg^2+^ solvation environment by partially regulating the coordination structure of Mg^2+^, weakening the strong Mg^2+^-DME interaction and facilitating Mg^2+^ desolvation during deposition. Meanwhile, the preferential decomposition of TEP-derived phosphorus-containing species contributes to the formation of a stable phosphate-rich interphase, thereby improving interfacial stability and Mg deposition reversibility. Therefore, the novelty of this work lies not merely in introducing TEP into a Mg electrolyte, but in demonstrating a synergistic solvation and interphase regulation strategy enabled by TEP in an OTf^–^/Cl^–^ dual-salt Mg electrolyte. This differs fundamentally from previous TEP-based electrolyte designs and provides new insights into the molecular-level regulation of Mg^2+^ coordination chemistry and electrode/electrolyte interfacial evolution.

In this work, we design a cost-effective electrolyte by incorporating an optimal amount of 10% TEP cosolvent into the baseline electrolyte. The results confirm that TEP achieves synergistic modulation of Mg^2+^ solvation configuration and anode interfacial behavior. It can replace DME molecules and coordinate with Mg^2+^, thus distancing Mg^2+^ from DME and softening the structural deformation of bound DME. Meanwhile, the organophosphorus molecules in the rearranged solvation sheath preferentially undergo reductive decomposition on the surface of the Mg metal anode, in situ forming a dense and robust phosphorus-containing SEI layer that suppresses electron transfer and promotes rapid Mg^2+^ migration. As a result, the Mg||Mg symmetric cell with the optimized electrolyte realizes stable and ultralong cycle life of over 3500 h with an overpotential below 0.2 V at 0.1 mA cm^–2^ and 0.05 mAh cm^–2^, and the average CE of Mg deposition/dissolution in Mg||Mo asymmetric cell is higher than 99.4% after 3000 cycles at 0.5 mA cm^–2^and 0.25 mAh cm^–2^. Importantly, the Mg||α-MnO_2_ full cells can work stably at a rate up to 0.5 C, and a discharge capacity of 176.9 mAh g^–1^ is retained after 350 cycles at 0.1 C. It is hoped that this study can provide new ideas and experimental basis for the development of high-performance, easily prepared, and high-stability magnesium battery electrolytes, and promote the practical process of RMBs.

## 2 Experimental Section

### 2.1 Electrolyte Preparation

Magnesium trifluoromethanesulfonate (Mg­(OTf)_2_, 99.5%), magnesium chloride (MgCl_2_, ≥ 98%), 1,2-dimethoxyethane (DME, 99.5%) and triethyl phosphate (TEP, ≥ 99.5%) were purchased from Sigma-Aldrich. Four Å molecular sieves was purchased from Aladdin. The α-MnO_2_ cathode employed in this work was a commercial electrode and was utilized directly without further modification. The mass loading of the α-MnO_2_ cathode was 0.987 mg cm^–2^.

Molecular sieves were oven-dried at 200 °C for 24 h, vacuum-dried overnight, and stored in an Ar-filled glovebox (Mikrouna, H_2_O < 0.1 ppm, and O_2_ < 1 ppm). DME was dried using molecular sieves for at least 24 h. The baseline electrolyte (0.3 M Mg­(OTf)_2_+0.2 M MgCl_2_-DME, denoted as 0% TEP or TEP-free electrolyte) was prepared as follows: 483.7 mg of Mg­(OTf)_2_ was dissolved in 2 mL of DME under thorough stirring for 30 min, followed by the addition of 95.2 mg of MgCl_2_ and continuous stirring at 45 °C for 1 h to yield the clear baseline electrolyte. Based on this formulation, a series of TEP-modified electrolytes with TEP volume fractions of 5%, 10%, 15%, 20%, 25%, 30%, 35% and 40% were prepared by adjusting the TEP volume fractions while maintaining the total electrolyte volume at 2 mL (denoted as 5% TEP, 10% TEP, 15% TEP, 20% TEP, 25% TEP, 30% TEP, 35% TEP, and 40% TEP, respectively). In addition, we also prepared other types of electrolytes as control samples for comparison following the same procedures as described above (denoted as 0.3 M Mg­(OTf)_2_+0.1 M MgCl_2_-DME+10% TEP and 0.3 M Mg­(OTf)_2_+0.3 M MgCl_2_-DME+10% TEP). All electrolytes were dried over 4 Å molecular sieves overnight prior to use.

### 2.2 Electrochemical Testing

Magnesium foil and working electrodes (Cu, Al, SS, Ti, Mo) were punched into circular disks with a diameter of 13 mm. Prior to cell assembly, magnesium foils were sequentially polished with 1500-grit and 3000-grit sandpapers to eliminate surface impurities and native passivation layers. All CR2032-type coin cells were assembled in the argon-filled glovebox with glass fiber separators (Whatman, GF/D), and sealed under a uniaxial pressure of 1.0 T. Electrochemical measurements were performed at room temperature (25 °C) using a LAND battery testing system (LAND-CT-2001A) and an electrochemical workstation (CHI760E A201011). To evaluate the Coulombic efficiency (CE), cyclic voltammetry (CV), and linear sweep voltammetry (LSV), half-cells were assembled using magnesium foil as the anode and various current collectors-including Cu, Al, SS, Mo, and Ti foils as the working electrodes. The charge cutoff voltage for CE measurements was set at 1.2 V. CV tests were carried out at a scan rate of 5 mV s^–1^, while LSV tests were performed at 5 mV s^–1^. Mg||Mg symmetric cells were fabricated using two magnesium foils with identical surface treatment for electrochemical impedance spectroscopy (EIS), limiting current, and galvanostatic charge–discharge tests. EIS measurements were implemented with an amplitude of 5 mV over a frequency range of 10^6^-10^–2^ Hz. For limiting current tests, the areal capacity was fixed at 0.2 mAh cm^–2^, and the current density was varied from 0.05 to 6 mA cm^–2^. Mg||MnO_2_ full cells were assembled using α-MnO_2_ as the cathode and magnesium foil as the anode.

### 2.3 Material Characterization

Fourier transform infrared spectroscopy (FT-IR) and Raman spectroscopy were utilized to characterize the chemical composition and molecular structure of the prepared electrolytes. For postcycling electrode characterization, residual glass fiber separators on the electrode surface were removed by soaking the electrodes in DME under argon protection. The surface morphology and microstructure of the electrodes were observed via scanning electron microscopy (SEM). X-ray diffraction (XRD) with a Cu–Kα radiation source was employed to analyze the surface composition of cycled Mg anodes, with a scanning range of 10–90° and a scanning rate of 10° min^–1^. X-ray photoelectron spectroscopy (XPS) was performed to investigate the chemical bonding states and species composition on the surface of cycled Mg anodes. The measurements were conducted under a vacuum of <5 × 10^–9^ Torr, and Ar^+^ ion sputtering was applied at a rate of 10 nm min^–1^. All XPS spectra were fitted using CasaXPS software, with the C 1s peak at 284.4 eV as the reference for binding energy calibration.

## 3 Results and Discussion

### 3.1 Physicochemical and Structural Properties of TEP-Modified Electrolytes

The solubility and ionic conductivity play a vital role in electrolyte solutions. Figure 1a illustrates the solubility of 0.3 M Mg­(OTf)_2_+0.2 M MgCl_2_-DME with various volume fractions of TEP. The optimization of the two salt concentrations was discussed later in the text. All the electrolytes remained homogeneous and transparent solutions. Compared with the TEP-free electrolyte, the TEP-containing electrolyte systems achieved complete dissolution within a shorter period, and the dissolution rate was further accelerated with increasing TEP content. This phenomenon demonstrates that TEP, acting as a strong Lewis base, effectively participates in the primary solvation sheath of Mg^2+^, disrupts undesirable ion-pair interactions, and facilitates full dissociation and dissolution of Mg salts. Figure 1b depicts the corresponding ionic conductivity of the electrolytes with different TEP fractions. The conductivity of the TEP-free electrolyte remained below 90 μS cm^–1^, which is ascribed to the poor dissociation of Mg salts and the predominance of contact ion pairs or larger aggregates that reduce the number of effective charge carriers. As the TEP content increased, the conductivity rose sharply, reaching a maximum of approximately 128–130 μS cm^–1^ at 10–15% TEP. This enhancement is attributed to the improved salt dissociation and the formation of solvent-separated ion pairs or free ions, facilitated by the coordination of TEP with Mg^2+^. However, when the TEP content exceeded 15%, the conductivity declined progressively. This decrease is due to increased viscosity and the formation of an overly thick solvation shell around Mg^2+^, which impedes ion mobility, or the reformation of neutral ion aggregates at high TEP concentrations.^25^

**Figure 1:**
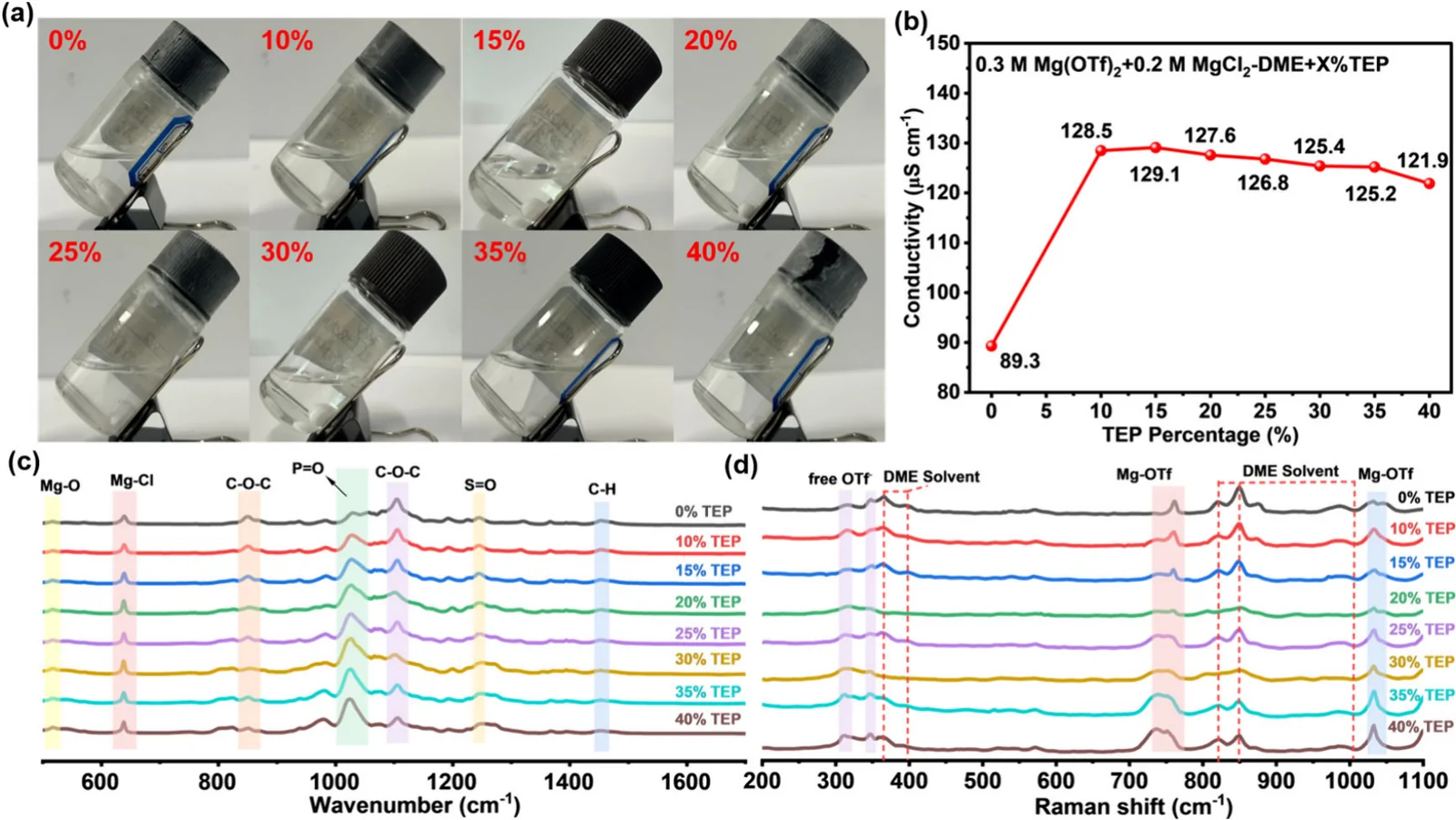
Physicochemical properties and structural characterizations of the TEP-free and TEP-modified electrolytes with varying TEP volume fractions. (a) Optical images, (b) Ionic conductivity, (c) FT-IR spectra and (d) Raman spectra.

To elucidate the influence of TEP as a cosolvent on regulating the solvation and coordination behavior of Mg^2+^ in magnesium-based electrolytes, we conducted a complementary FT-IR and Raman spectroscopic analysis of 0.3 M Mg­(OTf)_2_+0.2 M MgCl_2_-DME with various volume fractions of TEP (Figure 1c-d). FT-IR analysis first revealed key structural changes: FT-IR analysis first revealed key structural changes: the Mg–Cl vibrational peak at 638 cm^–1^ intensified and broadened with increasing TEP, indicating the formation of soluble mixed-ligand Mg–Cl complexes that mitigate precipitation and sustain mobile charge carriers. Concurrently, the stretching peak at 849 cm^–1^ of the C–O–C bond in DME red-shifted and weakened, signaling competitive displacement of DME from the Mg^2+^ primary solvation shell. The TEP-specific P=O bond shifted from 1032 cm^–1^ to 1023 cm^–1^ (red shift), confirming direct coordination of TEP to Mg^2+^. Meanwhile, the diagnostic S=O stretching vibration of OTf^–^ exhibits a distinct blueshift from 1244 to 1272 cm^–1^ upon TEP incorporation. This spectroscopic evolution corroborates that the competitive incorporation of TEP into the Mg^2+^ primary solvation sheath markedly weakens the electrostatic coupling between divalent Mg^2+^ cations and OTf^–^ anionic moieties. Such coordination competition further disrupts densely aggregated contact ion pairs and facilitates the dissociation of cation–anion clusters, thereby regulating the overall solvation configuration of the electrolyte system. Furthermore, Raman spectroscopy further revealed a progressive increase in the vibrational intensity of free OTf^–^ anions (≈350 cm^–1^), alongside diminished vibrational intensity signature (≈760 cm^–1^) of the [Mg^2+^-OTf^–^] contact ion pair (CIP), confirming TEP-promoted dissociation of Mg­(OTf)_2_ salts. The DME solvent breathing mode also red-shifted and decreased in intensity, corroborating the FT-IR-observed displacement of DME by TEP via P=O coordination. These spectroscopic results demonstrate that TEP acts as a structure-directing cosolvent in the baseline electrolyte, disrupting ion aggregates, rearranging the Mg^2+^ solvation shell, and favoring soluble Mg species. Meanwhile, Raman spectra provided further insight into the evolution of Mg coordination structures. In our 0.3 M Mg­(OTf)_2_+0.2 M MgCl_2_-DME+TEP electrolyte, the [Mg^2+^]:[Cl^–^] molar ratio is larger than 1, indicating that the chloride concentration is insufficient to promote the extensive formation of multinuclear chloride-bridged Mg complexes.^26^ This assumption was further supported by the Raman spectra, where no obvious characteristic bands associated with multinuclear Mg–Cl aggregates were observed in the 200–300 cm^–1^ region (Figure S1a). In addition, a characteristic band at approximately 876 cm^–1^ was observed (Figure S1b), which is consistent with the reported vibrational feature of Mg­(OTf)_2_(DME)_2_ in DME-based Mg­(OTf)_2_ electrolytes.^14^ Notably, the intensity of this band gradually decreases with increasing TEP concentration, indicating the progressive disruption of the relatively stable Mg­(OTf)_2_(DME)_2_ coordination structure. This observation further supports the above conclusion that TEP significantly weakens the electrostatic coupling between Mg^2+^ cations and OTf^–^ anions by competitively coordinating with Mg^2+^, thereby promoting the reconstruction of the Mg^2+^ solvation structure and the formation of TEP-containing solvated species, such as [Mg­(DME)_*x*_(TEP)_*y*_]^2+^. These results suggest that most Mg species remain in Mg^2+^/solvent-coordinated states, while only a small fraction participates in the formation of Mg–Cl-containing species, rather than forming highly condensed multinuclear Mg–Cl clusters. To clarify the possible electroactive Mg species in the electrolytes, representative literatures were compared in terms of electrolyte compositions, solvents, and the corresponding Mg species (Table S2). These studies demonstrate that Mg speciation is strongly dependent on the solvent coordination environment, electrolyte composition, and [Mg^2+^]:[Cl^–^] molar ratio.

### 3.2 Electrochemical Performance of TEP-Modified Electrolytes

To systematically investigate the influence of TEP content on the long-term cycling stability, Mg||Mg symmetric cells were assembled for galvanostatic cycling tests. As shown in Figure 2a, the cycling performance of the 10% TEP-modified electrolyte was even more stable than that of the TEP-free electrolyte (>0.2 V) and it exhibited a lower polarization voltage of approximately 0.12 V for over 3200 h at 0.1 mA cm^–2^ and 0.05 mAh cm^–2^. A high polarization voltage (>1 V) was observed in the initial cycles, which is due to the removal of native oxide/passivation adsorbed on the Mg metal surface, electrochemical removal of electrolyte impurities during early cycles, or that the initial

**Figure 2:**
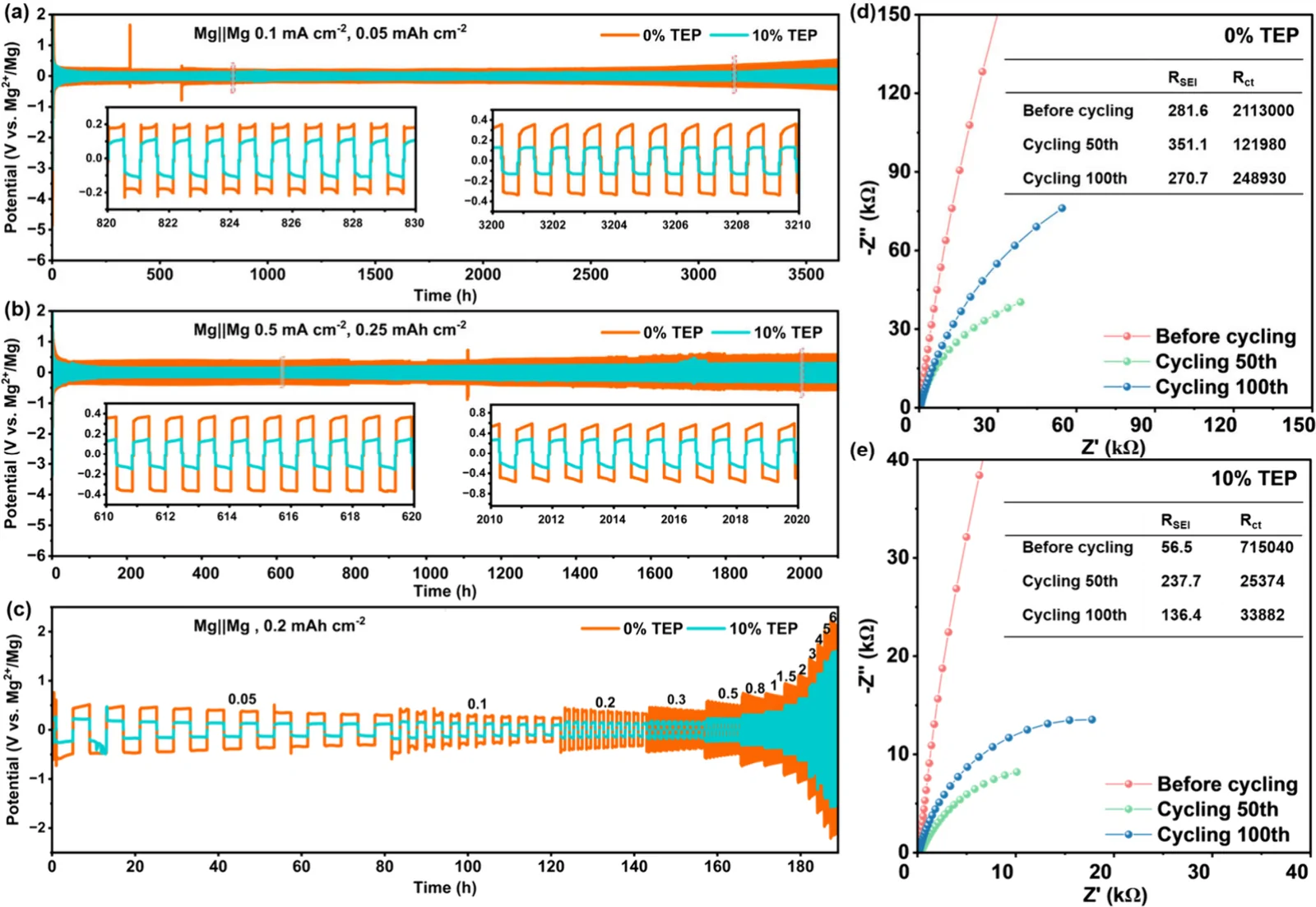
Electrochemical performance of Mg||Mg symmetric cells with the TEP-free and 10% TEP-modified electrolytes. (a) Long-cycle performance at 0.1 mA cm^–2^ and 0.05 mAh cm^–2^. (b) Long-cycle performance at 0.5 mA cm^–2^ and 0.25 mAh cm^–2^. (c) Rate performance at the current densities of 0.05–6 mA cm^–2^ (fixed areal capacity: 0.2 mAh cm^–2^). (d-e) EIS Nyquist plots of Mg||Mg symmetric cells cycled at 0.5 mA cm^–2^ and 0.25 mAh cm^–2^.

Mg deposition facilitates the nucleation of crystalline Mg in the successive cycles.^27–29^ The polarization voltage progressively decreased and remained stable without significant fluctuations in subsequent cycles, indicating the formation of an Mg^2+^-conductive SEI. At a higher current density of 0.5 mA cm^–2^ and 0.25 mAh cm^–2^, the TEP-free electrolyte exhibited a higher overpotential (>0.4 V) along with pronounced voltage fluctuations. In contrast, the 10% TEP-modified electrolyte showed a stable cycling performance with an overpotential of only approximately 0.22 V for more than 2000 h (Figure 2b). As further illustrated in Figure S2, S3 and S4, 5% TEP and 15% TEP electrolytes delivered superior cycling stability over the TEP-free electrolyte, while 20–40% TEP electrolytes led to high polarization voltage along with substantial fluctuations, since excess TEP coordinates with Mg^2+^, resulting in a decrease in the degree of ionic coordination, which yields an interphase with insufficient electronic insulation.^30^ Collectively, these results establish 10% TEP as the optimal electrolyte for balancing high ionic conductivity and long-term cycling stability. 10% TEP exhibited optimal performance, thus the TEP volume fractions and Mg­(OTf)_2_ concentration were fixed at 10% and 0.3 M, to systematically investigate the influence of MgCl_2_ concentration on the electrolyte system. The electrolyte with a lower-concentration MgCl_2_ (0.1 M) showed polarization fluctuations and then experienced severe short-circuiting at 650 h (Figure S5a). The electrolyte with 0.3 M MgCl_2_ exhibited a lower polarization voltage of approximately 0.13 V for 760 h at 0.1 mA cm^–2^ and 0.05 mAh cm^–2^, after which the overpotential quickly exceeded 0.5 V with further cycling (Figure S5b). Electrochemical evaluations demonstrated that all electrolytes with varied MgCl_2_ content delivered inferior long-term cycling stability and higher polarization in comparison with the 10% TEP, verifying that 0.2 M is the most favorable MgCl_2_ concentration for achieving superior electrochemical performance in the electrolyte system.

Due to the inherent nature of the electrochemical deposition process, dendritic structures typically form when a high current density is applied to deposit metals. The maximum current density limit that allows for dendrite-free Mg deposition is highly dependent on the electrolyte solution used.^31^ Thus, it is important to determine this current density limit to avoid the dendritic growth of Mg with our electrolyte system. Figure 2c presents the rate performance of Mg||Mg symmetric cells at current densities of 0.05–6 mA cm^–2^ with a fixed areal capacity of 0.2 mAh cm^–2^. For the TEP-free electrolyte, the overpotential increased precipitously from 0.4 V at 0.05 mA cm^–2^ to 2.3 V at 6 mA cm^–2^. In direct comparison, the 10% TEP-modified electrolyte exhibited substantially lower and more stable voltage polarization from 0.1 V at 0.05 mA cm^–2^ to 1.5 V at 6 mA cm^–2^ without soft short-circuiting, indicating a highly homogeneous and uniform deposition of Mg. We further evaluated the rate performance of the electrolytes with other various TEP volume fractions in Figure S6. The results revealed that all TEP-modified electrolytes maintained stable overpotentials across the tested current density range and exhibited relatively low polarization even under high-rate conditions. Notably, compared with the TEP-free electrolyte, the introduction of TEP significantly enhances the electrolyte’s tolerance to high current densities, thereby improving its overall electrochemical stability under demanding operating conditions.

The critical role of the TEP cosolvent in regulating the interfacial kinetics of the Mg metal anodes was further explored by EIS studies on Mg||Mg symmetric cells at various cycles, including the pristine state before cycling and after the 50th and 100th cycles. In Nyquist plots, the resistance corresponding to the intersection with the horizontal axis is the Ohmic resistance (*R*_*s*_), and the semicircular region is the overall interface resistance (*R*_*int*_), including interphase resistance (*R*_*SEI*_) and charge-transfer resistance (*R*_*ct*_).^32–34^ The results after fitting through the equivalent circuit (Figure S7 and Table S1) were shown in Figure 2d-e. Large interfacial impedance in the Nyquist plots was observed in cells with the TEP-free and 10% TEP-modified electrolytes before cycling, which corresponds to the large overpotential in the initial cycle and indicates that the surface of the uncycled Mg anode is covered by a dense native passivation layer that severely hinders efficient Mg^2+^ transport. In subsequent cycles, the TEP-free electrolyte maintained remarkably high interfacial resistances during cycling (*R*_*SEI*_ = 270.7 Ω at the 100th cycle). However, the *R*_*SEI*_ of 10% TEP-modified electrolyte at 50th and 100th cycles was only 237.7 and 136.4 Ω, respectively, indicating the gradual formation and stabilization of an ionically conductive interphase. Meanwhile, the 5%, 15%, 20%, 25%, 30%, 35% and 40% TEP-modified electrolytes also exhibited discernible semicircles with low impedance postcycling (Figure S8a-g), indicating that TEP effectively regulates interfacial reactions and promotes the formation of a stable SEI with improved Mg^2+^ transport kinetics.

The Mg deposition/dissolution behavior of the TEP-free and 10% TEP-modified electrolytes on the Mo working electrode is demonstrated by CV measurements (Figure 3a-b). The CV curves were obtained after 50–100 charge/discharge conditioning cycles, during which the Mg||Mo cell underwent an initial activation process and subsequently exhibited stable and conditioning-free Mg deposition/dissolution behavior with highly overlapping voltammograms. To provide further insight into this activation process, the CEs and corresponding charge/discharge voltage profiles obtained during the precycling activation process, together with the CV curves recorded during the initial four cycles were provided in Figure S9. Both electrolytes underwent an initial activation process followed by gradual stabilization of the electrochemical response. Notably, the voltage hysteresis decreased from 1.04 V for the 0% TEP electrolyte to 0.74 V for the 10% TEP electrolyte, indicating reduced polarization and improved Mg plating/stripping kinetics upon TEP incorporation. Furthermore, the 10% TEP-modified electrolyte displayed well-defined Mg deposition/dissolution profiles, and the CV curves recorded at different cycle numbers exhibited substantial overlap with no obvious current decay or peak shift, indicating that the electrolyte can sustain stable Mg deposition/dissolution processes on Mo working electrode with good electrochemical reversibility. The initial overpotential for Mg deposition was low, approximately −0.15 to −0.20 V, suggesting a small nucleation barrier for Mg on the Mo substrate. Meanwhile, the onset potential for Mg dissolution was located near 0 V, implying low polarization during the dissolution process. Figure S10 presents the CV tests of asymmetric cells with10% TEP-modified electrolyte on other working electrodes (Al, Cu, SS, and Ti), all of which exhibited a strong current response and a highly reversible Mg deposition/dissolution behavior. In addition, CV measurements were further conducted on the electrolytes with other TEP volume fractions using different working electrodes (Al, Cu, SS, Ti and Mo). For all TEP-containing electrolyte systems, the CV profiles consistently demonstrated a progressive enhancement in cycling stability and electrochemical reversibility with successive cycling, indicative of continuous electrode activation and improved Mg deposition/dissolution kinetics during cycling. Notably, among all working electrodes evaluated, the Mo working electrode demonstrated the most superior cycling consistency and electrochemical reversibility across all TEP-containing electrolyte systems, with highly overlapping CV curves and minimal peak potential shift over consecutive cycles (Figure S11, S12, S13, S14 and S15). The anodic stability of the TEP-modified electrolytes on various current collectors was further studied by LSV measurements. For the TEP-free electrolyte (Figure 3c), the anodic current density increased sharply at <3.5 V on all tested current collectors, indicating the onset of electrolyte decomposition or side reactions. In striking contrast, the 10% TEP-modified electrolyte (Figure 3d) delivered a substantially enhanced anodic stability across the entire range of current collectors. On a Mo working electrode, the electrolyte exhibited an astonishing anodic stability of up to 4.5 V. Practically, the electrolyte exhibited sufficiently high anodic stability versus various non-noble metals. The anodic stability was beyond 4 V on Al, Cu, SS, and Ti electrodes. Figure S16 displays the LSV curves of other electrolytes with varying TEP volume fractions on multiple working electrode substrates. The results consistently demonstrated that all TEP-containing electrolytes exhibited substantially higher oxidative stability (>3.5 V) on every current collector compared to the TEP-free baseline electrolyte. These findings collectively corroborate that the incorporation of TEP effectively enhances the anodic stability of the dual-salt electrolyte, thereby broadening its electrochemical stability window and enabling compatibility with high-voltage cathode materials for high-performance magnesium batteries.

**Figure 3:**
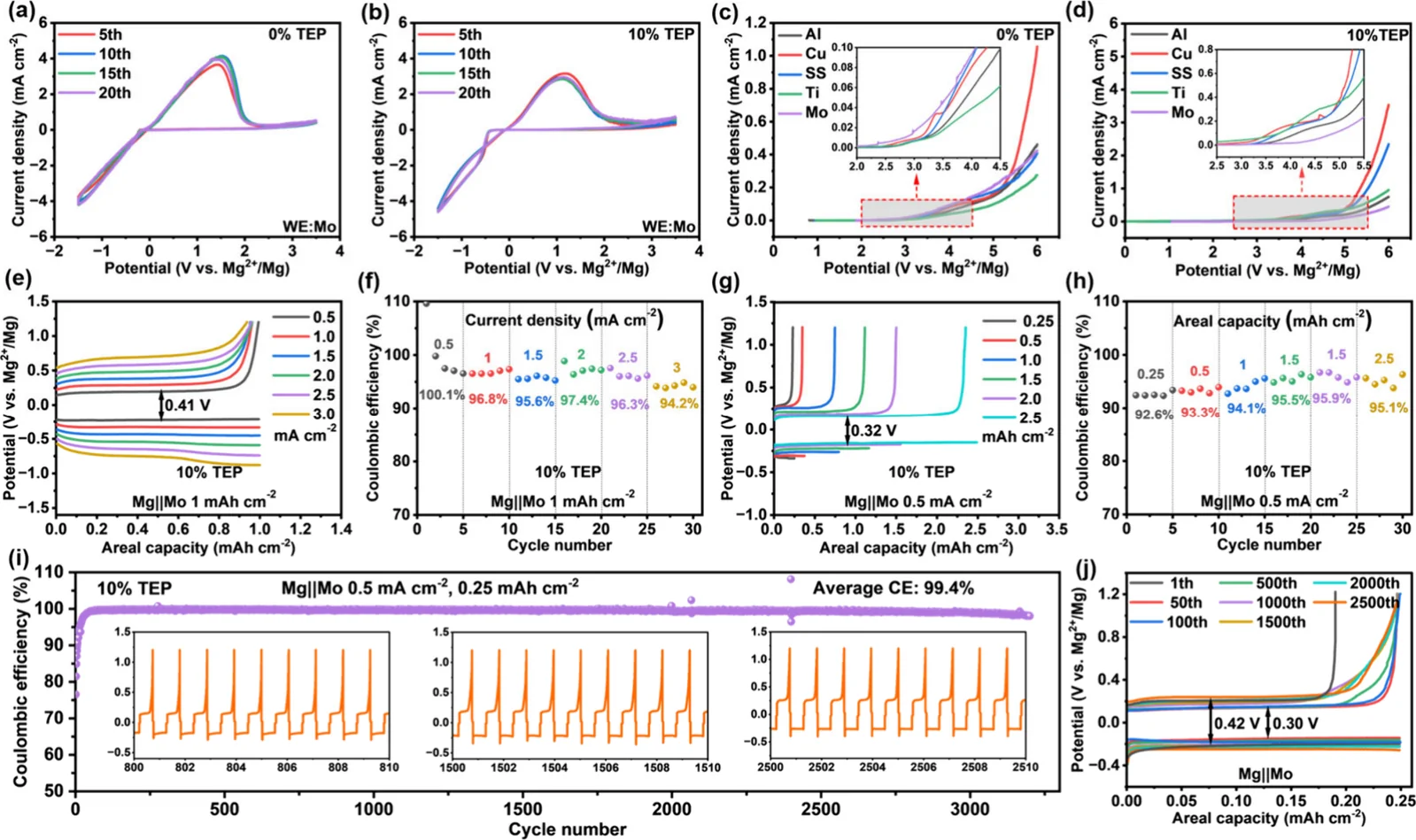
Electrochemical performance of Mg||Mo asymmetric cells with the TEP-free and 10% TEP-modified electrolytes. (a-b) Typical CV curves at a scan rate of 5 mV s^–1^ in a potential range of −1.5–3.5 V. (c-d) LSV curves using different working electrodes (Al, Cu, SS, Ti, Mo) at a scan rate of 5 mV s^–1^. (e) Voltage profiles and (f) CEs of 10% TEP-modified electrolyte at the current density of 0.5–3 mA cm^–2^ with fixed areal capacity of 1 mAh cm^–2^. (g) Voltage profiles and (h) CEs of 10% TEP-modified electrolyte at the areal capacity of 0.25–2.5 mAh cm^–2^ with a fixed current density of 0.5 mA cm^–2^. (i) CE and (j) voltage profiles of 10% TEP-modified electrolyte at 0.5 mA cm^–2^ and 0.25 mAh cm^–2^.

The galvanostatic cycling performance of asymmetric cells was also examined under various cycling conditions to provide a useful benchmark for evaluating electrolyte performance. Homogeneous metal deposition/dissolution at high areal capacity and high current density is crucial for achieving high energy and high-power densities of Mg batteries toward commercial applications. Figure 3e-f present the voltage profiles and CEs of the Mg||Mo asymmetric cell in 10% TEP electrolyte at current densities ranging from 0.5 to 3 mA cm^–2^ while keeping constant areal capacity at 1 mAh cm^–2^. It should be noted that deposition at high current densities will accelerate dendritic growth, which will lead to decreased CE and internal short-circuiting. The overpotentials of Mg deposition at current densities of 0.5, 1, 1.5, 2, 2.5, and 3 mA cm^–2^ were −0.22 V, −0.32 V, −0.43 V, −0.54 V, −0.65 V and −0.76 V, respectively. Despite increased overpotentials, high CE was maintained between 94.2%–100.1% at all current densities, indicating excellent reversibility of Mg deposition/dissolution. Toward high-energy applications, we also examined the reversibility of Mg deposition and dissolution at high areal capacity while keeping the current density at 0.5 mA cm^–2^ (Figure 3g-h). At areal capacities of 0.25, 0.5, 1.0, 1.5, 2.0, and 2.5 mAh cm^–2^, the CEs were 92.6%, 93.3%, 94.1%, 95.5%, 95.9%, and 95.1%, respectively. The marginally lower CE at high areal capacities is probably due to the formation of electrically isolated Mg crystals caused by the nonuniform dissolution process of thick Mg deposits.

To evaluate the reversibility of Mg deposition/dissolution, the long-term cycling tests of the Mg||Mo asymmetric cell were conducted at 0.5 mA cm^–2^ and 0.25 mAh cm^–2^. The first Mg dissolution showed a low overpotential at 0.21 V and delivered the initial CE of 76.6%. The irreversible capacity corresponds to the reduction of trace moisture and impurities present in the electrolyte and cell components. The CE was 84.9% in the second cycle, reached 99.1% by the 40th cycle, and stabilized at 99% over 3000 cycles (Figure 3i). Meanwhile, the Mg deposition and dissolution overpotentials were reduced and well maintained near ± 0.15 V (Figure 3j), indicating remarkably stable cycling and a typical behavior of a conditioning-free electrolyte system. Furthermore, all the working electrodes were extended to comprehensively evaluate the CEs and voltage curves of electrolytes with varying TEP volume fractions. The results demonstrated that 5–15% TEP exhibited stable CEs and low polarization voltages on all working electrodes (Figure S17, S18, S19), indicative of excellent Mg deposition/dissolution reversibility and minimal parasitic side reactions. However, as the TEP content was further increased (20–40% TEP), both the CEs decreased drastically and the polarization voltage increased significantly simultaneously (Figure S20, S21, S22, S23, and S24). This performance degradation can be attributed to the remarkably increased viscosity of the electrolyte at high TEP concentrations, which impedes Mg^2+^ migration and desolvation kinetics, thereby raising the interfacial resistance and overpotential. Meanwhile, excessive TEP also induces the formation of a thicker and less conductive SEI layer on the electrode surface, which further hinders charge transfer and gives rise to aggravated polarization and deteriorated reversibility. These results demonstrate that moderate TEP content incorporated into the baseline electrolyte is indispensable for simultaneously achieving balanced interfacial stability, low polarization, and high CE. Excess TEP dosage can enable fast interfacial charge transfer and improved ion conductivity in bulk electrolytes at the expense of oxidation stability and Mg deposition/dissolution stability. Therefore, reasonable regulation of TEP content to balance these fundamental electrochemical properties is critical for realizing stable and high-efficiency electrochemical reactions.

### 3.3 Mg Metal Anode Interfacial SEI Characterizations and Mechanisms

To further explore the significant influence of the TEP cosolvent on the deposition morphology, SEM characterization was conducted on the Mg electrode surfaces of Mg||Mg symmetric cells after 100 cycles. The uneven deposition of Mg under high current density and the severe pitting behavior under moderate current density in Cl-containing electrolytes have become critical degradation mechanisms of Mg anodes.^35^ As shown in Figure 4a-b, the Mg anode cycled in the TEP-free electrolyte exhibited a severely corroded, rough surface with numerous irregular protrusions, cracks, and loose, powdery byproducts, indicative of uncontrolled interfacial side reactions and Mg dendrite growth. The cross-sectional view further revealed a thick, uneven corrosion layer with a maximum thickness of ≈52.9 μm (Figure 4c), confirming severe parasitic reactions and structural degradation of the Mg anode in the TEP-free system. In sharp contrast, the Mg anode cycled in the 10% TEP-modified electrolyte displayed a smooth, dense, and defect-free surface with no obvious dendrites or corrosion products (Figure 4d-e), demonstrating effective suppression of interfacial side reactions and uniform Mg deposition/dissolution behavior. The cross-sectional analysis confirmed the formation of a thin, compact, and uniform protective layer with a thickness of only ≈26.3 μm (Figure 4f). Notably, as displayed in Figure S25, the surface morphology evolution of cycled Mg anodes presented distinct concentration-dependent characteristics along with the rising TEP proportion. With 15% TEP, the Mg anode maintained a relatively intact and smooth surface, with only sporadic tiny corrosion spots and mild localized degradation behaviors. As the TEP volume fraction gradually increased to 20% and 25%, the size and quantity of aggregated corrosion products on the Mg surface continued to expand, accompanied by gradually aggravated surface roughening and localized pit corrosion. Once the TEP volume fraction was further elevated to 30% and 35%, severe interfacial corrosion became increasingly prominent. Large-scale agglomerated corrosive deposits extensively covered the Mg anode surface, accompanied by obvious structural damage and uneven Mg deposition behavior. Particularly for the high-concentration 40% TEP system, the Mg anode suffered catastrophic interfacial corrosion, presenting abundant scattered broken corrosion debris, serious surface collapse and disordered parasitic deposits across the entire electrode surface. These results clearly demonstrate that excessive TEP content breaks the optimized synergistic regulation effect on electrode–electrolyte interfaces. Oversaturated TEP molecules disrupt the stable Mg^2+^ solvation sheath, trigger uncontrollable continuous parasitic reactions at the anode interface, accelerate severe corrosion degradation of metallic Mg, and induce chaotic Mg deposition/dissolution behaviors. Consequently, excessively high TEP proportion severely deteriorates interfacial stability, greatly impairs the long-term cycling reversibility of Mg anodes, and ultimately weakens the overall electrochemical performance of magnesium batteries, which well explains the performance degradation of high-concentration TEP electrolytes in electrochemical tests.

**Figure 4:**
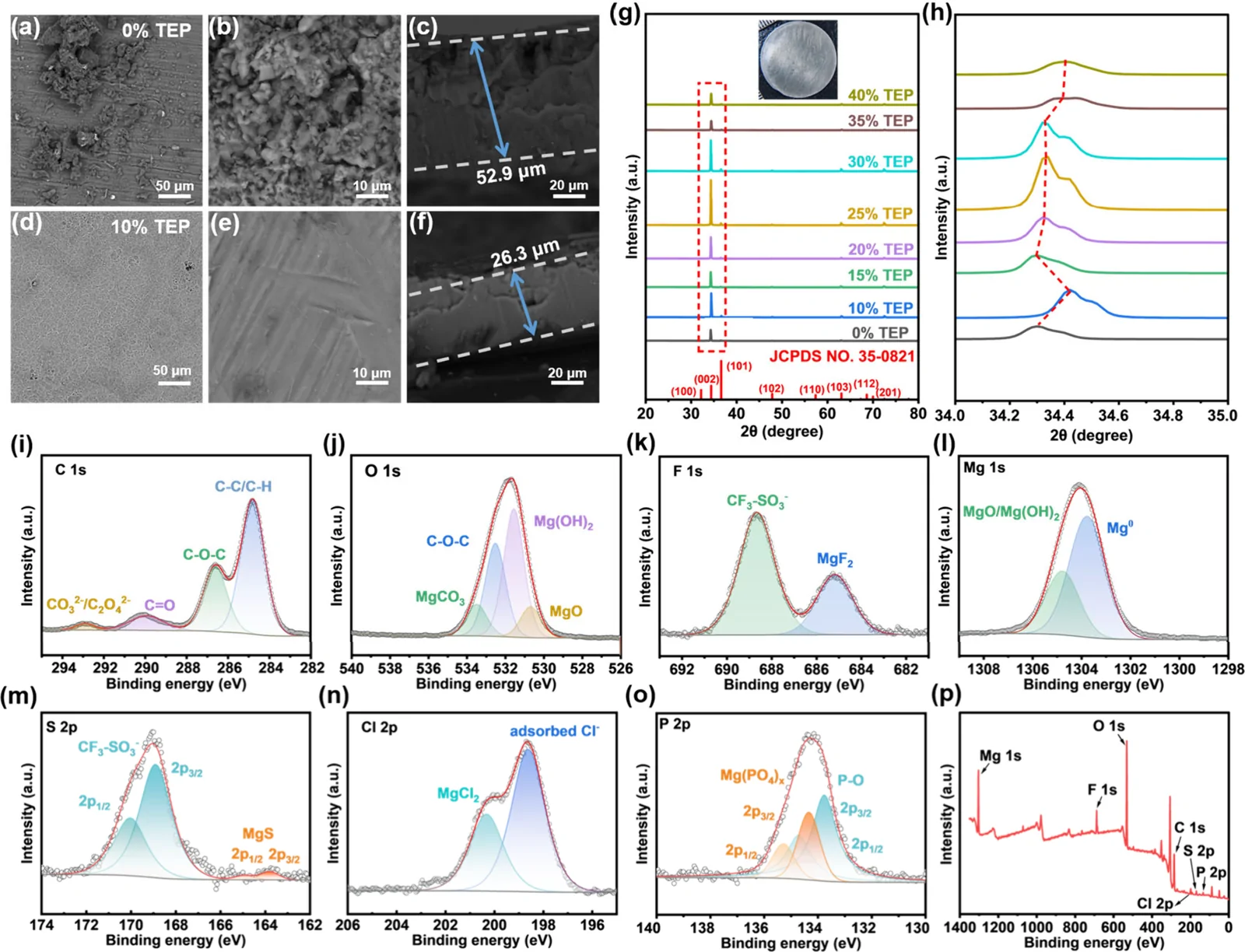
Morphology and structural characterization of Mg deposition from TEP-free and TEP-modified electrolytes. Top view and cross-section SEM images of Mg deposition after 100 cycles in the TEP-free (a-c) and 10% TEP-modified (d-f) electrolytes. (g-h) XRD patterns of Mg deposition after 100 cycles in the electrolytes with varying TEP concentrations. XPS spectra of the deposition on Mg anodes after 100 cycles in 10% TEP-modified electrolyte: (i) C 1s, (j) O 1s, (k) F 1s, (l) Mg 1s, (m) S 2p, (n) Cl 2p and (o) P 2p spectra (p) survey spectra.

The crystal structure and phase composition of the cycled Mg anodes were further analyzed by XRD. The main diffraction peaks are well-indexed to the P63/mmc space group of hexagonal Mg (JCPDS No.35–0821),^36^ with no additional impurity peaks detected, confirming the high purity of the Mg substrate (Figure 4g). However, significant variations in peak intensity and sharpness were observed among different electrolytes. The strongest diffraction peak in all electrolyte systems corresponds to the (002) crystal plane of metallic magnesium, located at approximately 2θ≈34.4°. In the 10% TEP-modified electrolyte, the (002) diffraction peak exhibited the most prominent intensity and the sharpest profile, indicating that the magnesium anode delivered the highest crystallinity and a well-ordered lattice arrangement. Nevertheless, pronounced low-angle shifts of characteristic diffraction peaks were clearly observed for the TEP-free electrolyte and high-concentration TEP electrolytes (Figure 4h). With the gradual deviation of TEP content from the optimal 10% TEP, the diffraction peaks continuously shifted toward smaller 2θ angles, accompanied by gradual peak broadening and intensity attenuation. Such evolution manifests a progressive increase in interplanar spacing, which represents evident lattice expansion of the magnesium matrix. Consequently, the diffraction peaks of all nonoptimal electrolytes shift markedly to lower 2θ angles. Insufficient TEP fails to disassemble tight ion pairs and homogenize deposition behavior, while excessive TEP triggers deteriorated interfacial evolution and irreversible lattice strain. Both improper TEP dosages lead to inferior structural stability of the magnesium anode, whereas the well-tailored solvation and interfacial structure in the 10% TEP system effectively suppress lattice distortion and maintain stable crystalline characteristics.

The chemical composition of the Mg anode-electrolyte interface is crucial to the reversibility and kinetics of the Mg deposition/dissolution processes.^37,38^ XPS was used to investigate SEI compositions on the plated Mg surface with 10% TEP-modified electrolyte in an Mg||Mg asymmetric cell after 100 cycles at 0.5 mA cm^–2^ and 0.25 mAh cm^–2^. As shown in Figure 4i-o, the C 1s, O 1s, F 1s, Mg 1s, S 2p, P 2p and Cl 2p XPS spectra demonstrate that a SEI containing organic and inorganic ingredients is formed on Mg electrodes after cycling. The C 1s spectrum contains peaks at C–C/C–H (284.8 eV), C–O–C (286.5 eV), C=O (290.2 eV), and CO_3_^2–^/C_2_O_4_^2–^ (292.8 eV).^39,40^ For the O 1s spectrum, distinct peaks assignable to MgO (530.3 eV), Mg­(OH)_2_ (531.6 eV), C–O–C (532.8 eV), and MgCO_3_ (533.7 eV) are identified.^41–43^ Combined with the C 1s and O 1s results, it can be concluded that the surface of the cycled Mg anode contains abundant organic and inorganic species derived from the decomposition of ether-based solvents. MgF_2_ (685.3 eV) originates from the decomposition of OTf^–^,^14^ which is the only source of fluorine in the electrolyte solution. In addition, a strong signal at ≈688.5 eV (CF_3_–SO_3_^–^) in the F 1 s spectrum occurs due to the presence of residual OTf^–^.^14,44^ The Mg 1s spectrum further confirmed the SEI composition, with the main peak at ≈1302.8 eV corresponding to metallic Mg and the shoulder peak at ≈1304.7 eV (MgO/Mg­(OH)_2_) derived from the decomposition of DME and the influence of trace H_2_O. In the S 2p spectrum, MgS is detected with a peak at ≈163.7 eV, which originates from the reduction of OTf^–^.^45^ The peak from 198.5 to 200.5 eV in the Cl 2p spectrum suggests the presence of chloride species, which includes MgCl_2_ (200.4 eV) and adsorbed/complexed Cl^–^ (198.5 eV).^46,47^ Notably, the P 2p spectrum exhibited two well-resolved peaks at ≈133.8 eV (P–O) and ≈134.5 eV (Mg-PO_4_^3–^),^23,48^ which are characteristic of phosphate species derived from the preferential reduction of TEP. The survey XPS spectrum further confirms that TEP actively participates in SEI formation (Figure 4p), generating a Mg­(PO_4_)_*x*_-rich inorganic layer that acts as a robust barrier helping to block electrons and facilitate Mg^2+^ transport.

According to the above results, the relationship between the solvation sheath and the SEI structure is clarified in Figure 5. TEP facilitates the in situ construction of a dense, uniform, and ion-conductive SEI rich in phosphate-derived inorganic components, which suppresses excessive decomposition of OTf^–^ anions and DME, mitigates Mg corrosion and dendrite formation, and stabilizes the electrode/electrolyte interface during prolonged cycling. In sharp contrast, the baseline electrolyte without TEP forms a thick, heterogeneous, and resistive SEI that impedes ion transport and results in rapid performance fading. The distinct difference in SEI composition and structure originates from the fundamental regulation of TEP on the solvation structure and interfacial reaction pathways. In the TEP-free electrolyte, Mg^2+^ preferentially coordinates with DME molecules to form a stable solvation sheath dominated by DME, in which this solvation structure exhibits strong Mg^2+^-solvent interactions, leading to sluggish desolvation kinetics and a high energy barrier for Mg^2+^ to release from the solvent cage. Concurrently, continuous parasitic reactions of the electrolyte components (DME, OTf^–^) at the Mg anode surface yield a disordered, uneven, and defective SEI film primarily composed of insulating phases such as MgO, MgF_2_, and MgCl_2_. This heterogeneous SEI severely impedes Mg^2+^ transport across the interface, resulting in high polarization voltage and poor cycling stability. Upon introducing TEP, the solvation structure and interfacial behavior are significantly modulated. TEP competes with DME for Mg^2+^ coordination via the electron-rich oxygen atoms in its P=O groups, partially replacing DME in the primary solvation shell and forming phosphorus-containing active solvation species. This structural reconstruction weakens Mg^2+^-solvent interactions, markedly reducing the desolvation energy barrier and enabling rapid desolvation at the electrode interface. Meanwhile, the organic phosphorus species in the modified solvation sheath undergo preferential decomposition on the Mg anode surface, inducing the formation of a uniform, compact, and Mg^2+^-conductive SEI layer enriched with Mg–P–O species (e.g., Mg_3_(PO_4_)_*x*_). This robust SEI effectively suppresses the excessive decomposition of DME and OTf^–^ anions, alleviates anode passivation, and provides a low-resistance pathway for Mg^2+^ transport. Consequently, highly reversible Mg deposition/dissolution behavior and excellent long-cycle stability are achieved.

**Figure 5:**
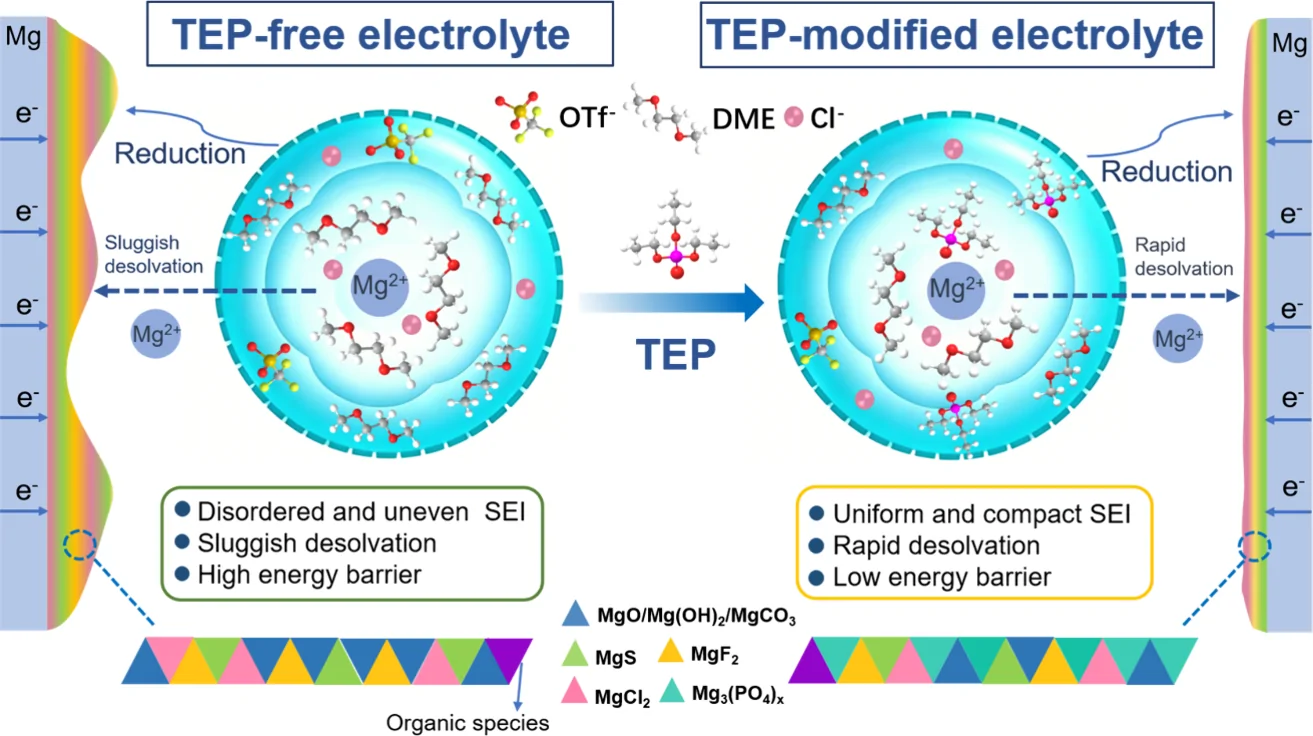
Schematic diagrams of the Mg^2+^ ions solvation sheath and SEI components on the Mg anode.

### 3.4 Electrochemical Performance of Mg||α-MnO_2_ Full Cells

To comprehensively evaluate the practical application of the TEP-modified electrolyte, Mg||α-MnO_2_ full cells were assembled and tested under various conditions. The full cell assembled with the TEP-free electrolyte only delivers a discharge capacity of 90.9 mAh g^–1^ after 350 cycles at 0.1C (1C = 308 mA g^–1^). In comparison, the Mg||α-MnO_2_ full cells with 10% TEP electrolyte demonstrated a reversible capacity of 176.9 mA g^–1^ and stable cycling performance after 350 cycles with a capacity retention of 64.5% at 0.1 C (Figure 6a-c). Meanwhile, as shown in Figure S26, the 10% TEP electrolyte exhibited relatively large initial discharge capacities of 353.3 mAh g^–1^, which exceeds the theoretical capacity of α-MnO_2_ (308 mAh g^–1^), commonly attributed to SEI formation.^49^ In addition, the 10% TEP electrolyte exhibited a gradually increasing CE with cycling and relatively smaller fluctuations compared with the 0% TEP electrolyte. Furthermore, its discharge capacity progressively stabilized and reached a relatively stable level after approximately the seventh cycle. In contrast, the 0% TEP electrolyte showed more pronounced capacity fluctuations during the initial cycles before gradually reaching a stable state. These results suggest that the incorporation of TEP facilitates a more gradual and stable electrochemical activation process, which is consistent with the formation and stabilization of a more stable electrode/electrolyte interphase. The differential capacity (dQ/dV) analysis further revealed the redox polarization and long-term kinetic stability of cells cycled in TEP-free and TEP-modified electrolytes. The potential separation between the cathodic reduction peak and anodic oxidation peak directly reflects voltage hysteresis and interfacial reaction kinetics. Although the dQ/dV profiles of both electrolytes maintained satisfactory peak overlap over 300 cycles at 0.1C, the 10% TEP electrolyte delivered a much smaller potential separation between redox peaks and significantly higher intensity (Figure S27a-b), indicating that TEP greatly accelerates Mg^2+^ interfacial transport and stabilizes long-term redox kinetics. The high-rate long-term cycling performance of the Mg||α-MnO_2_ full cells was further investigated at 0.5C (Figure 6d-f). The discharge capacity gradually decayed from 90.6 mAh g^–1^ at the first cycle to 52.4 mAh g^–1^ after 3000 cycles with a capacity retention of 57.8%. However, the TEP-free electrolyte only reached a maximum capacity of 35.4 mAh g^–1^ and then showed a gradual declining trend, highlighting the critical role of TEP in stabilizing the interface and accelerating Mg^2+^ transport kinetics at high rates. Importantly, the dQ/dV analysis after 2000 cycles showed well-preserved redox peaks without obvious fading, confirming that the TEP additive effectively suppresses interfacial side reactions and maintains fast ion transport kinetics compared with the severely disappeared redox peaks in the TEP-free electrolyte (Figure S27c-d).

**Figure 6:**
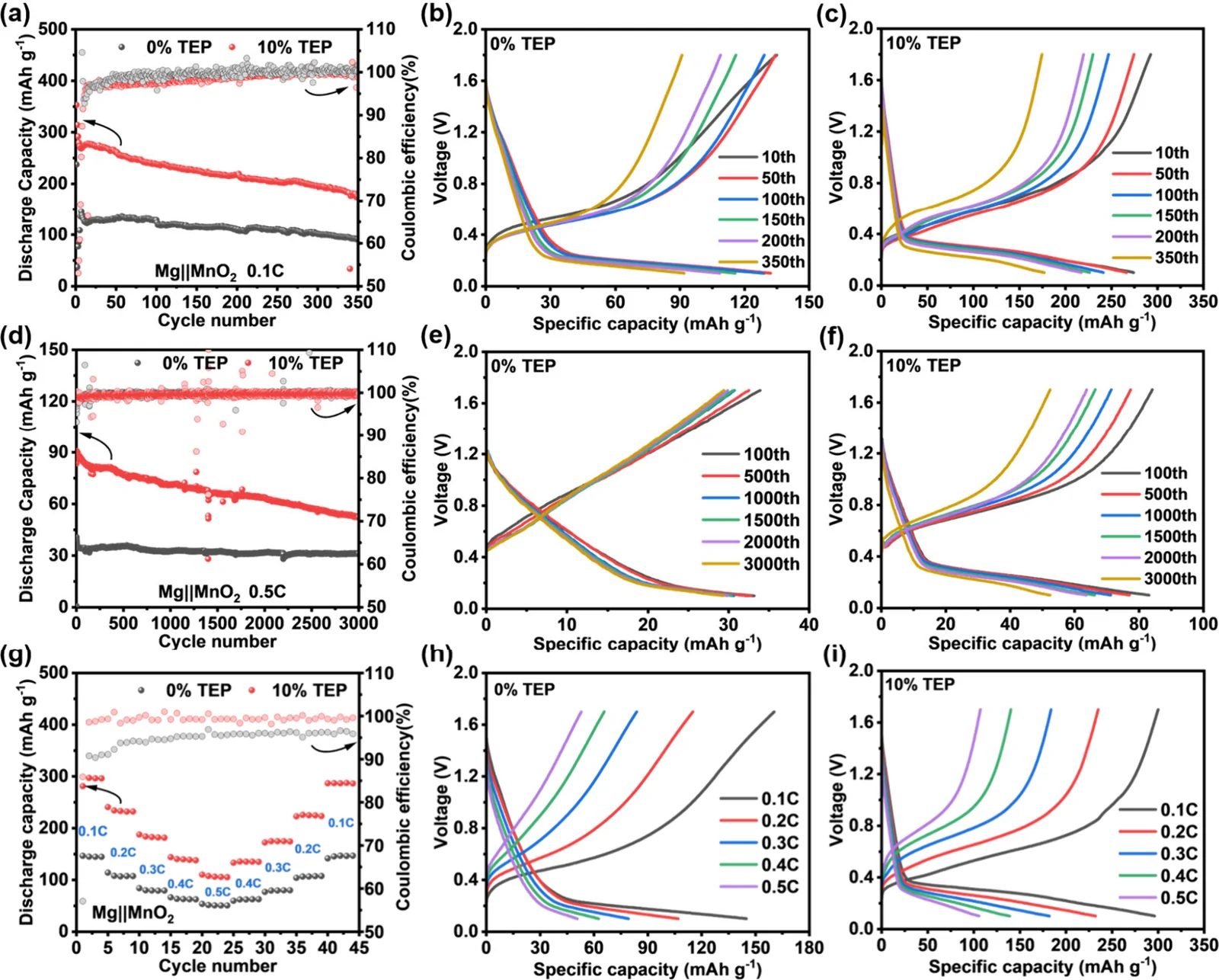
Electrochemical performance of Mg||α-MnO_2_ full cells with the TEP-Free and 10% TEP-modified electrolytes. (a) Long-term cycling performance at 0.1C and (b-c) corresponding voltage profiles. (d) Long-term cycling performance at 0.5C and (e-f) corresponding voltage profiles. (g) Rate performance from 0.1C to 0.5C and (h-i) corresponding voltage profiles.

The Mg||α-MnO_2_ full cell using TEP-modified electrolyte showed desirable rate performance (Figure 6g-i). The full cells delivered reversible discharge capacities of 296.2, 232.1, 182.0, 138.8, and 105.7 mAh g^–1^ at 0.1, 0.2, 0.3, 0.4, and 0.5 C, respectively, significantly outperforming the TEP-free electrolyte (144.9, 106.9, 79.2, 62.6, and 50.7 mAh g^–1^ at 0.1, 0.2, 0.3, 0.4, and 0.5 C, respectively). Notably, when the current density was returned from 0.5 to 0.1 C, the discharge capacity recovered to a level close to its initial value (286.9 mAh g^–1^), demonstrating excellent reversibility and structural stability. To further elucidate the kinetic superiority of the TEP-modified electrolyte under varying rates, rate-dependent differential capacity (dQ/dV) analysis was conducted. For the TEP-free electrolyte, as the current density increased, the dQ/dV curves exhibited severe peak broadening, a marked decrease in peak intensity, and a significant increase in the voltage gap between redox peaks (Figure S27e). This phenomenon indicates a substantial decline in electrochemical reaction efficiency, sluggish Mg^2+^ transport kinetics, and aggravated interfacial polarization at elevated rates. In sharp contrast, the TEP-modified cells maintained well-defined redox peaks with stable intensity and a consistently small voltage gap across all tested rates. At the rate from 0.1C to 0.5C, the dQ/dV peaks of the TEP-modified cell showed only slight broadening without obvious intensity decay, demonstrating that TEP can effectively optimize the electrode–electrolyte interface, accelerate ion transport, and maintain high reaction efficiency even under high-rate discharge conditions (Figure S27f).

## 4 Conclusions

In summary, systematic investigations into TEP concentration effects reveal that the 10% TEP yields the most favorable compatibility with the Mg electrode. As for the regulatory mechanism of TEP cosolvent, FT-IR and Raman results demonstrate that TEP can replace partial DME solvents and OTf^–^ anions around Mg^2+^, thereby reducing CIPs and optimizing the solvation structure. Furthermore, XPS analysis verified that the phosphides (e.g., Mg_3_(PO_4_)_2_) derived from TEP are preferentially reduced and decomposed on the Mg anode surface, which in situ induces the formation of a dense, uniform and phosphorus-rich SEI layer. This modified interfacial film possesses favorable Mg^2+^ conductivity, which can effectively inhibit the continuous decomposition of DME solvent and free OTf^–^ anions, suppress harmful side reactions, alleviate anode surface passivation, and provide stable Mg^2+^ transport channels. The 10% TEP-modified electrolyte can achieve stable and ultralong cycle life in the Mg||Mg symmetric cell over 3500 h at 0.1 mA cm^–2^ and 0.05 mAh cm^–2^, and the high average CE of Mg deposition/dissolution in the Mg||Mo asymmetric cell over 99.4% after 3000 cycles at 0.5 mA cm^–2^ and 0.25 mAh cm^–2^. It also enables uniform Mg deposition/dissolution behaviors at high current density (6 mA cm^–2^) with area capacity (0.2 mAh cm^–2^). Importantly, the compatibility of TEP-modified electrolytes was demonstrated successfully in a rechargeable Mg||α-MnO_2_ cell with the specific discharge capacity of 176.9 mA g^–1^ after 350 cycles at 0.1 C, along with outstanding rate capability. This work emphasizes the significance of the coordination strategy of Mg salts and solvents as well as cationic/anionic solvation in the investigation of RMBs.

## Supplementary Material



## Data Availability

The data that support the findings of this study are available on request from the corresponding author, XG Li, upon reasonable request.

## Notes

Raman spectra of TEP-free and TEP-modified electrolytes with varying TEP volume fractions; long-term cycling and rate performance of Mg||Mg symmetric cells with varying TEP volume fractions, current densities, and areal capacities; electrochemical performance of Mg||Mg symmetric cells with varying MgCl_2_ concentrations; equivalent circuits and fitted parameters for electrochemical impedance spectroscopy of 0% TEP and 10% TEP electrolytes; Nyquist plots of Mg||Mg symmetric cells with varying TEP volume fractions; initial activation behavior of Mg||Mo asymmetric cells with 0% TEP and 10% TEP electrolytes, including CEs, voltage profiles, and CV curves of the initial four cycles; electrochemical characterization of Mg-based asymmetric cells with different TEP volume fractions and working electrodes, including CV, LSV, CE, and voltage profiles; SEM characterization of Mg deposits after cycling; initial cycling behavior and dQ/dV analysis of Mg||α-MnO_2_ full cells; and a comparison of reported electroactive Mg species in representative ether-based Mg electrolytes (PDF)
