# Supplementary material for: Triethyl Phosphate Enables Regulated Solvation Structure and Robust Anode Interphase in Dual-Salt Mg(OTf)2‑MgCl2 Electrolytes for Rechargeable Magnesium Batteries
Source: ACS Cent Sci. 2026 Sep 7;12(9):1526–38. doi: 10.1021/acscentsci.6c01164 (PMC13614046; doi:10.1021/acscentsci.6c01164)
Supplement: Supplementary file 1 [file oc6c01164_si_001.pdf]

Supporting Information

Triethyl Phosphate Enables Regulated Solvation Structure and Robust Anode Interphase in Dual-Salt $Mg(OTf)_2$-$MgCl_2$ Electrolytes for Rechargeable Magnesium Batteries

Yan Liu[a], Li Qian[a], Xiaogang Li[a], *, Yuqing Tian[a], Jiaping Ma[a], Siyu Song[a], Yixian Zhang[a], Aihua Yuan[a], *, Huajun Tian[b], *

[a] School of Environmental and Chemical Engineering, Jiangsu University of Science and Technology, Zhenjiang 212100, PR China

E-mail: *lxgchina2056@just.edu.cn; aihua.yuan@just.edu.cn*

[b] Key Laboratory of Power Station Energy Transfer Conversion and System of Ministry of Education and Beijing Laboratory of New Energy Storage Technology, School of Energy Power and Mechanical Engineering, North China Electric Power University, Beijing, 102206, China

E-mail: *Huajun.Tian@ncepu.edu.cn*

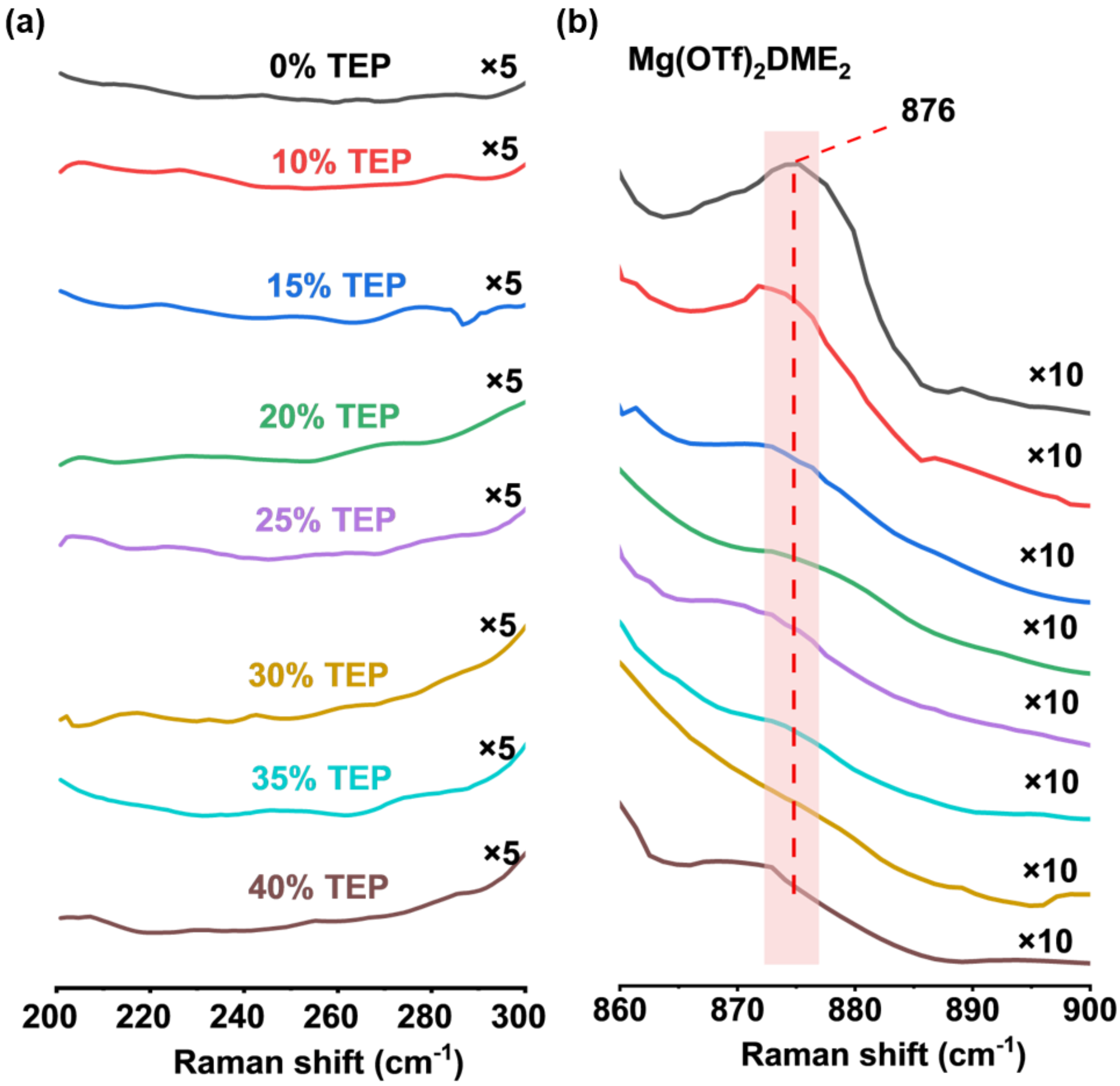

Figure S1. Raman spectra of the TEP-free and TEP-modified electrolytes with varying TEP volume fractions: (a) 200-300 cm^{-1}, (b)860-900 cm^{-1}.

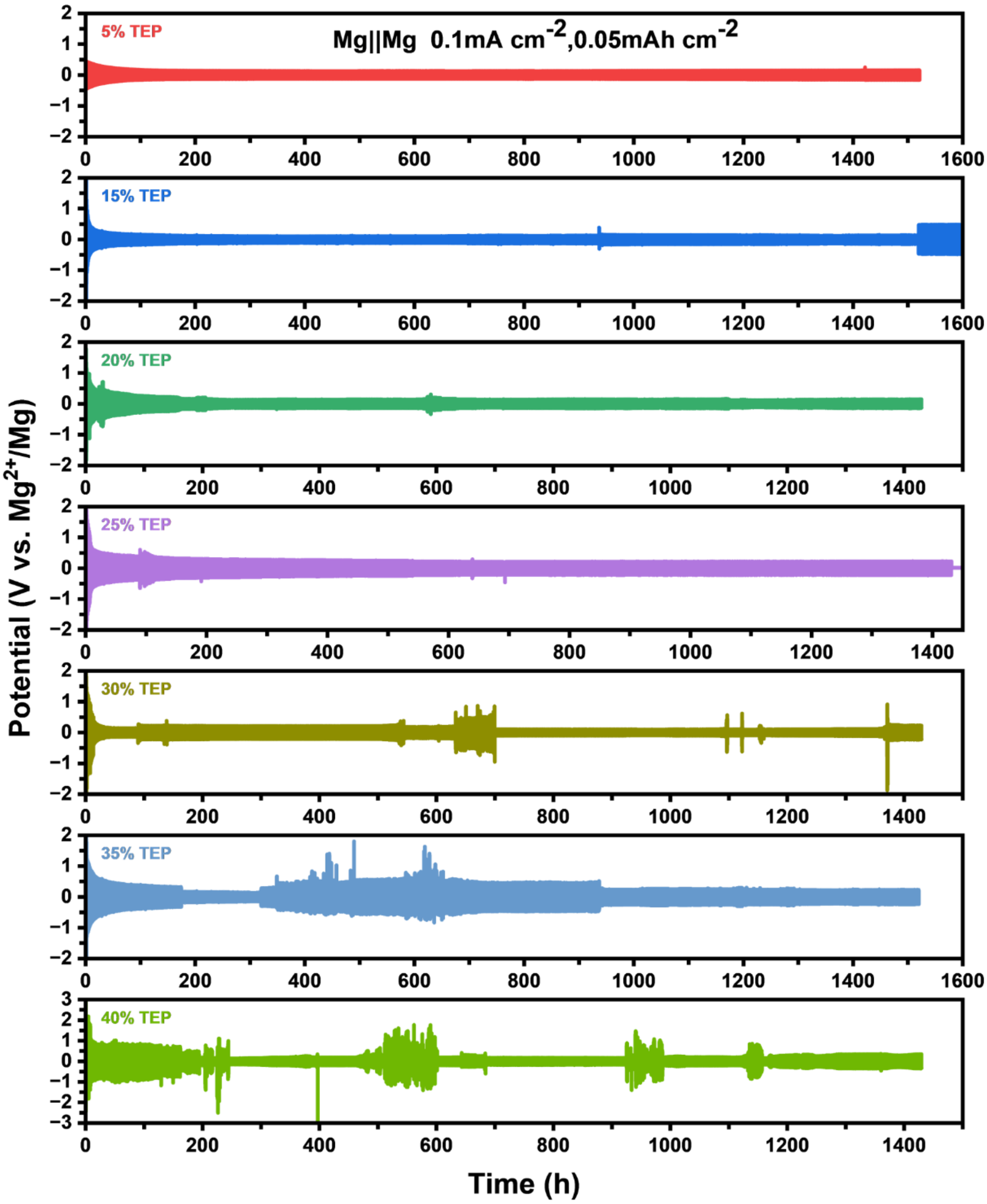

Figure S2. Long-term cycling performance of Mg||Mg symmetric cells in 0.3 M $Mg(OTf)_2$+0.2 M $MgCl_2$-DME electrolytes with varying TEP volume fractions at 0.1 mA cm^{-2} and 0.05 mAh cm^{-2}.

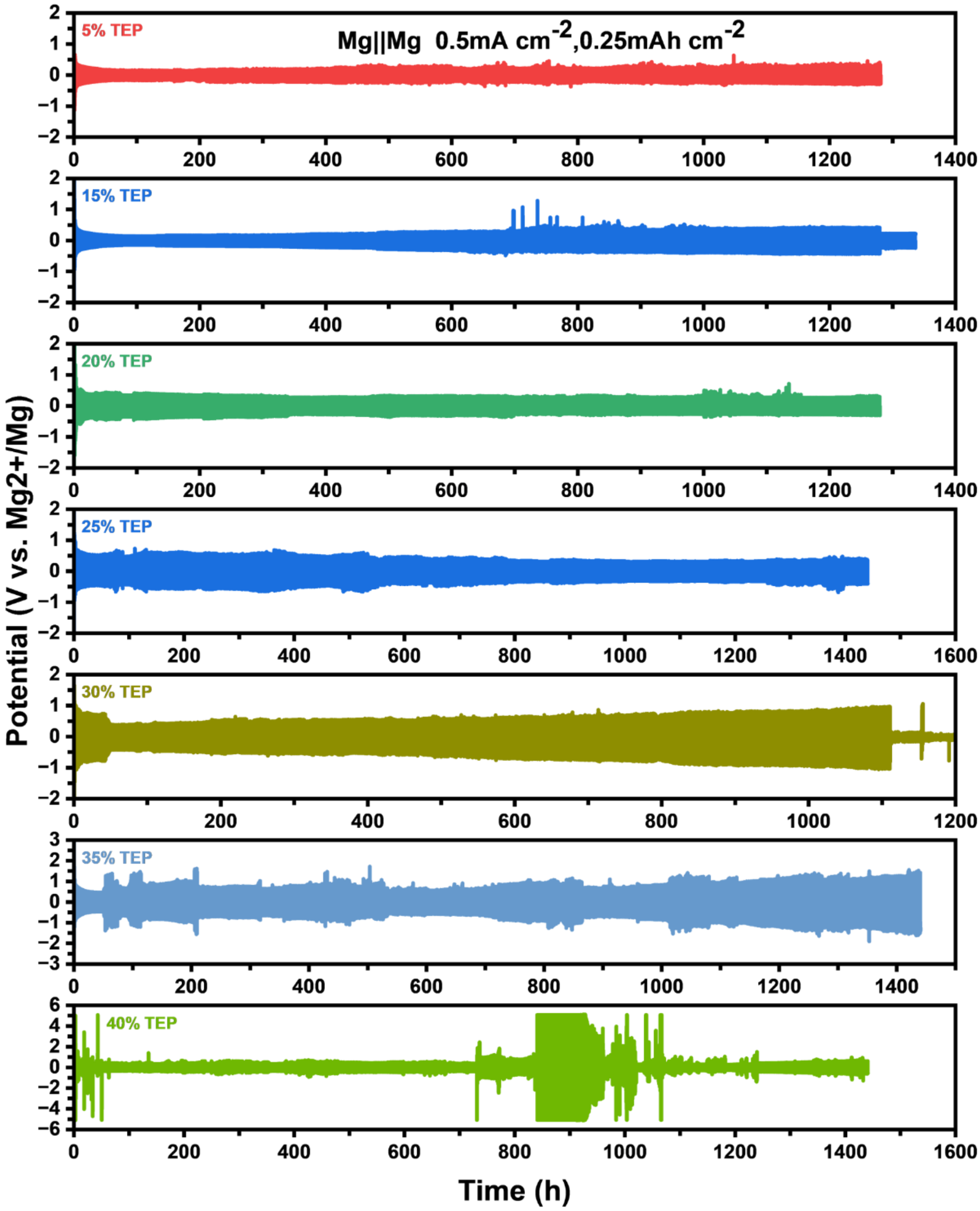

Figure S3. Long-term cycling performance of Mg||Mg symmetric cells in 0.3 M $Mg(OTf)_2$+0.2 M $MgCl_2$-DME electrolytes with varying TEP volume fractions at 0.5 mA cm^{-2} and 0.25 mAh cm^{-2}.

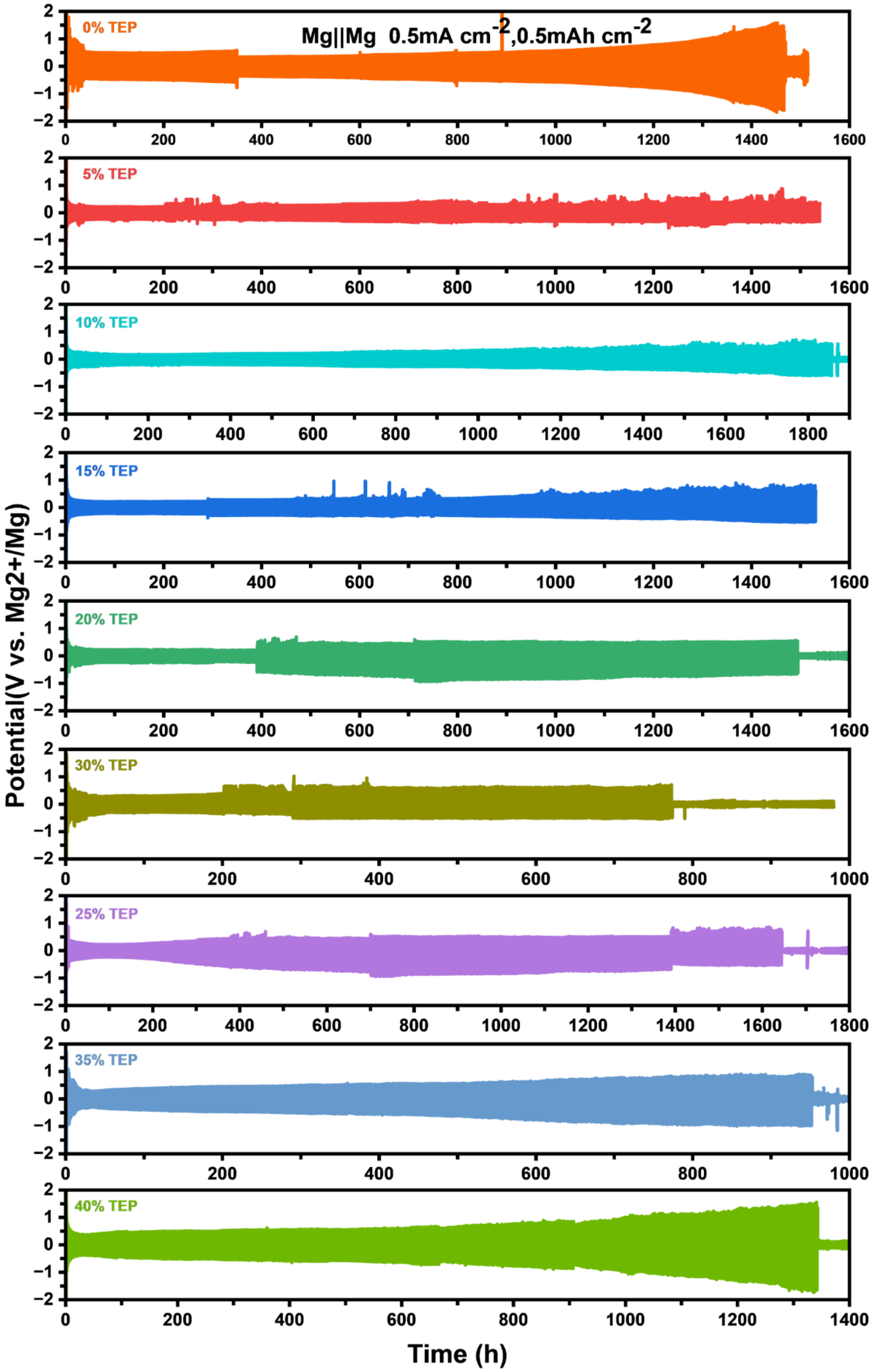

Figure S4. Long-term cycling performance of Mg||Mg symmetric cells in 0.3 M $Mg(OTf)_2$+0.2 M $MgCl_2$-DME electrolytes with varying TEP volume fractions at 0.5 mA cm^{-2} and 0.5 mAh cm^{-2}.

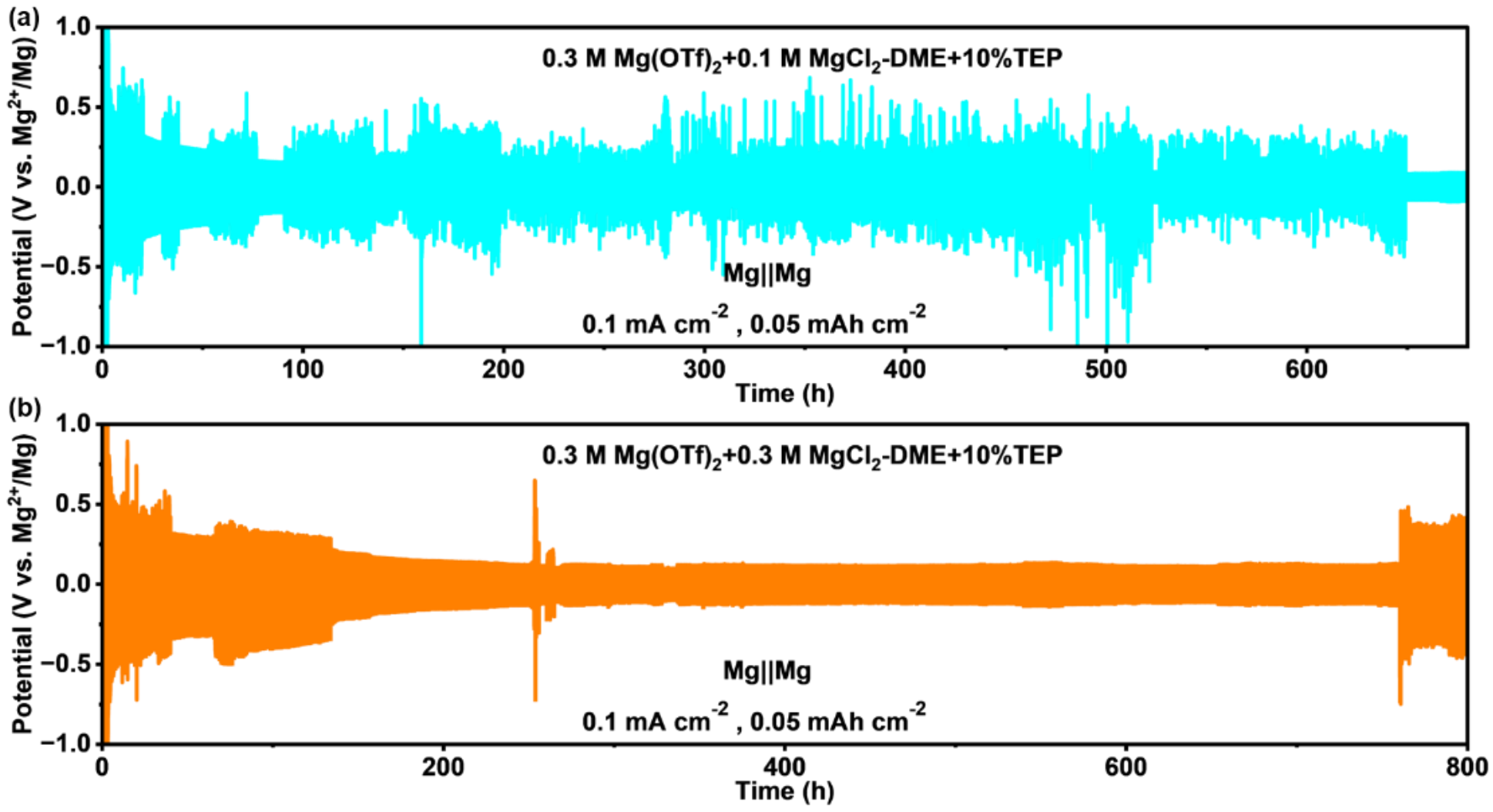

Figure S5. Long-term cycling performance of Mg||Mg symmetric cells with varying electrolytes. (a) 0.3 M $Mg(OTf)_2$+0.1 M $MgCl_2$-DME+10% TEP. (b) 0.3 M $Mg(OTf)_2$+0.3 M $MgCl_2$-DME+10% TEP.

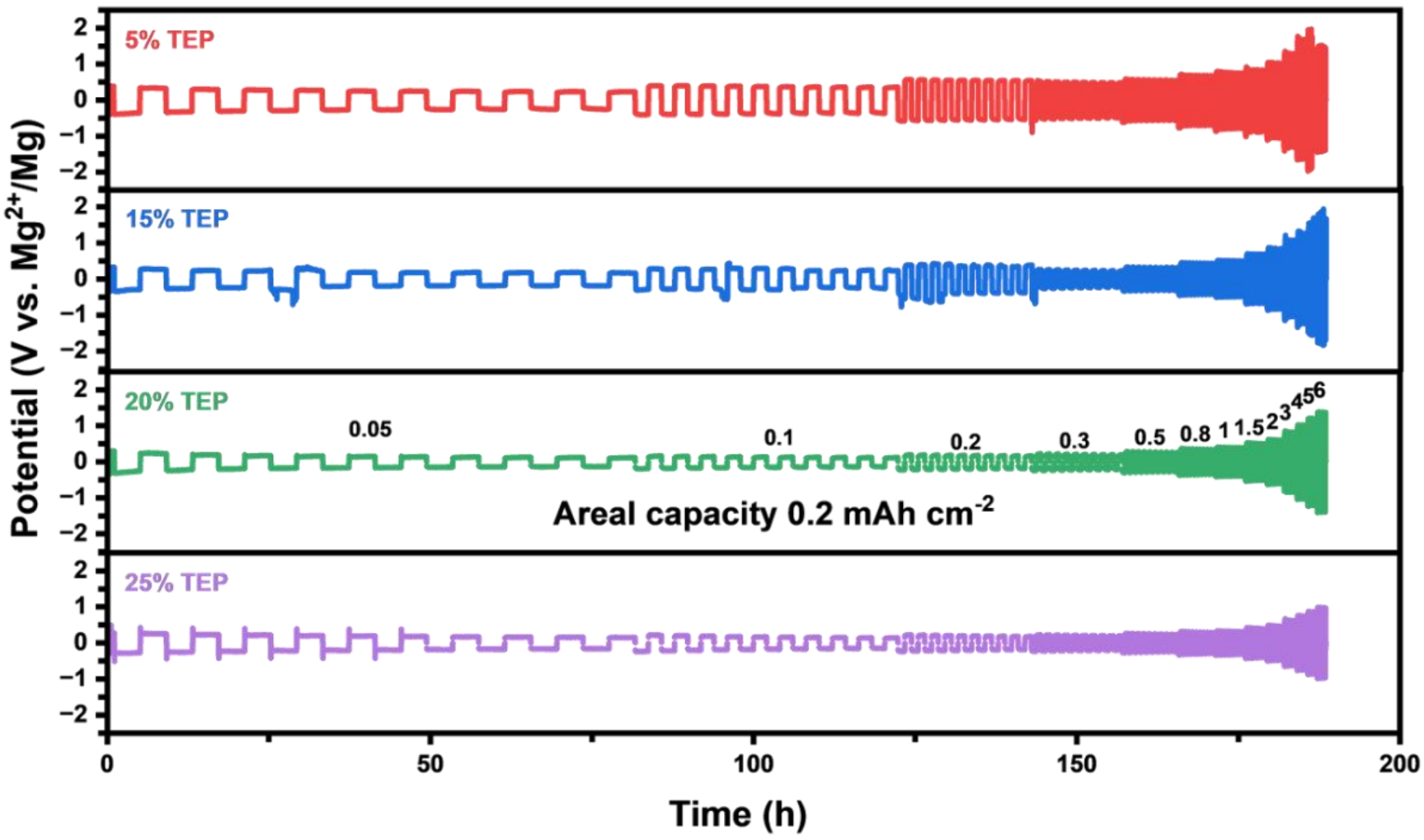

Figure S6. Rate performance of Mg||Mg symmetric cells in 0.3 M $Mg(OTf)_2$+0.2 M $MgCl_2$-DME electrolytes with varying TEP volume fractions at 0.05-6 mA cm^{-2} (fixed areal capacity: 0.2 mAh cm^{-2}).

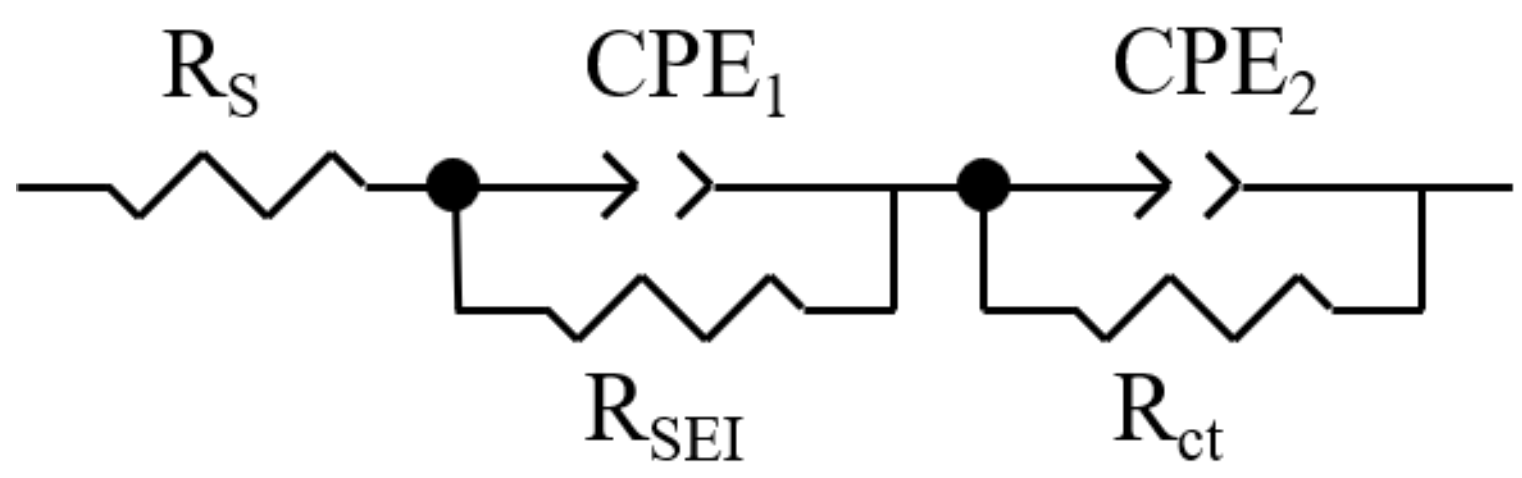

Figure S7. The equivalent circuits employed to analyze the EIS curves of the Mg||Mg cells with TEP-free and 10% TEP-modified electrolytes at the different cycles, in which R_s indicates the ohmic resistance, CPE is the constant phase element; R_{SEI} is corresponding to the resistance of ion migration in SEI, R_{ct} stands for the charge transfer resistance.

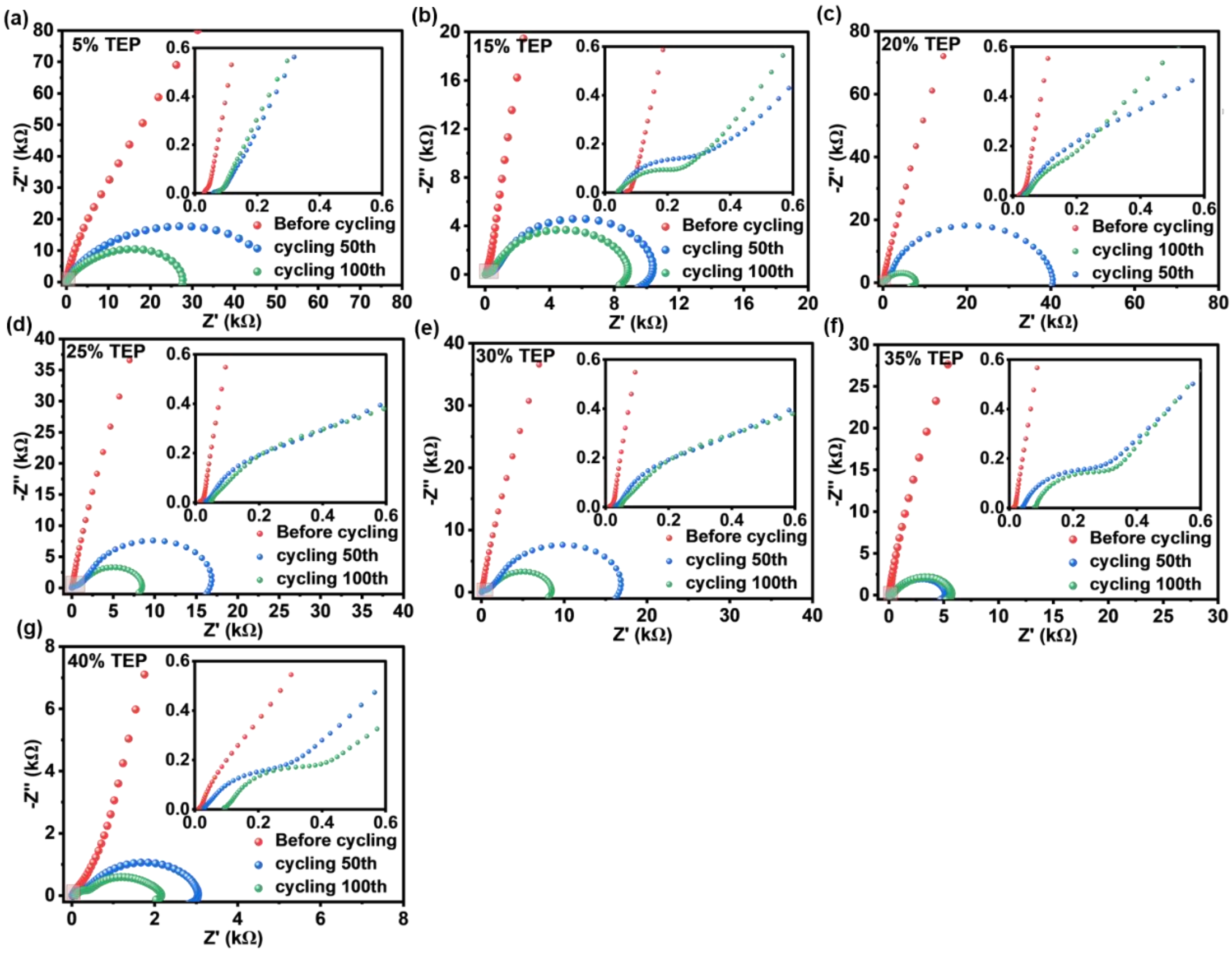

Figure S8. Nyquist plots of Mg||Mg symmetric cells in 0.3 M $Mg(OTf)_2$+0.2 M $MgCl_2$-DME electrolytes with varying TEP volume fractions at 0.5 mA cm^{-2} and 0. 25 mAh cm^{-2}: (a) 5% TEP, (b) 15% TEP, (c) 20% TEP, (d) 25% TEP, (e) 30% TEP, (f) 35% TEP, (g) 40% TEP.

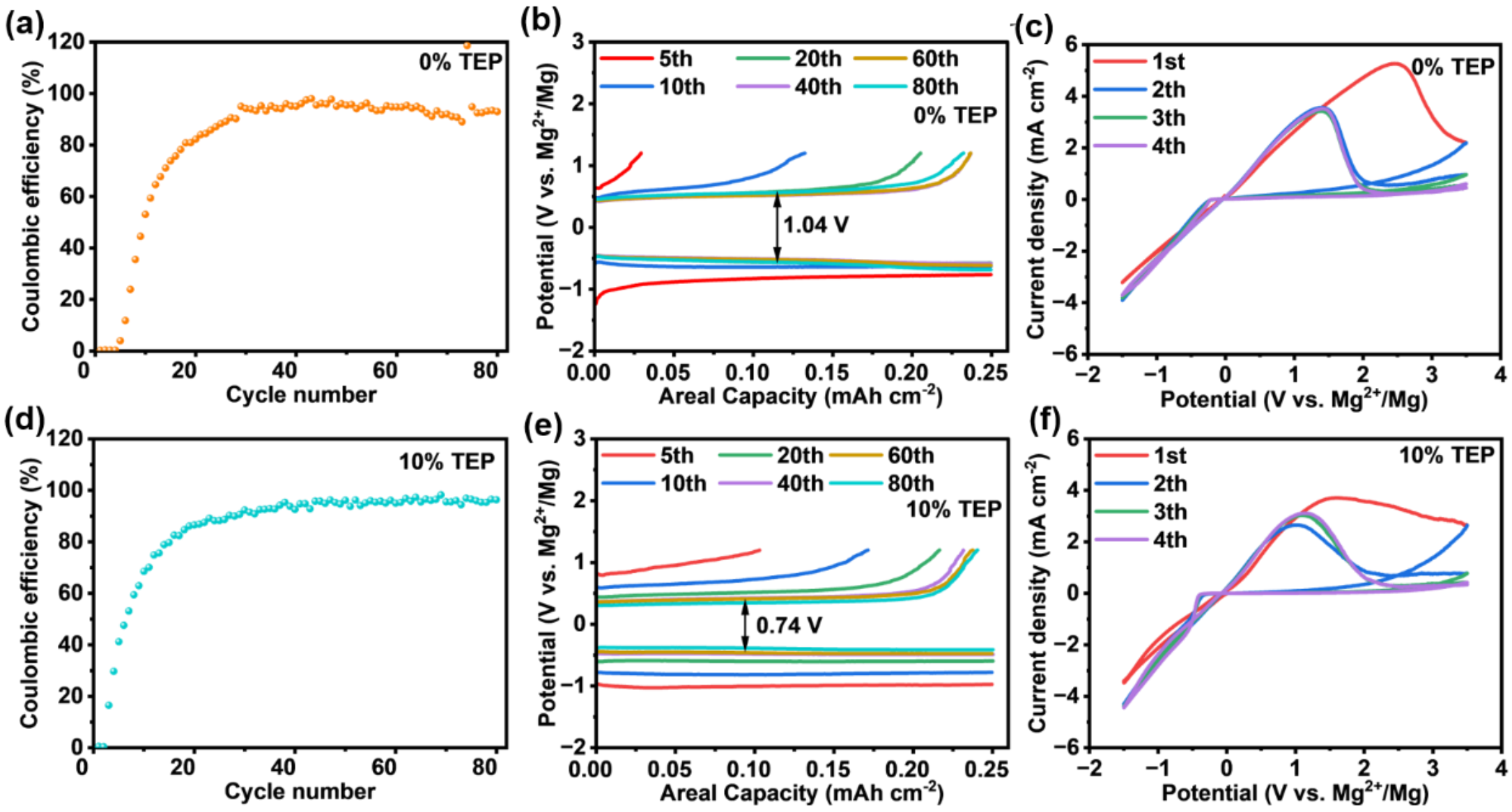

Figure S9. CEs and corresponding charge/discharge voltage profiles during the initial activation cycles, together with CV curves recorded during the initial four cycles, for (a-c) 0% TEP and (d-f) 10% TEP electrolytes.

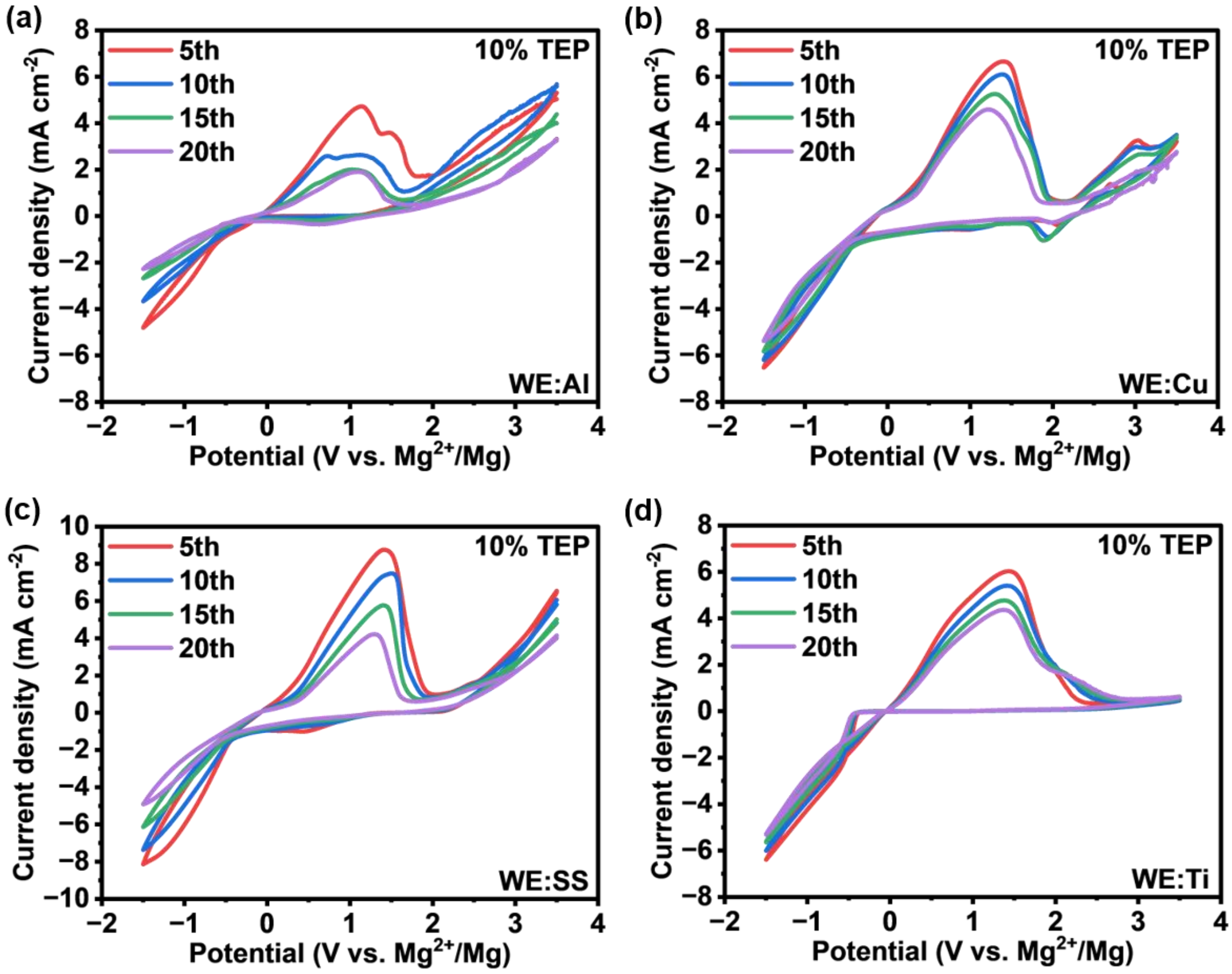

Figure S10. CV curves of Mg-based asymmetric cells in 0.3 M $Mg(OTf)_2$+0.2 M $MgCl_2$-DME electrolytes with 10% TEP using different working electrodes at a scan rate of 5 mV s^{-1} in a potential range of -1.5-3.5 V: (a) Al, (b) Cu, (c) SS, (d) Ti.

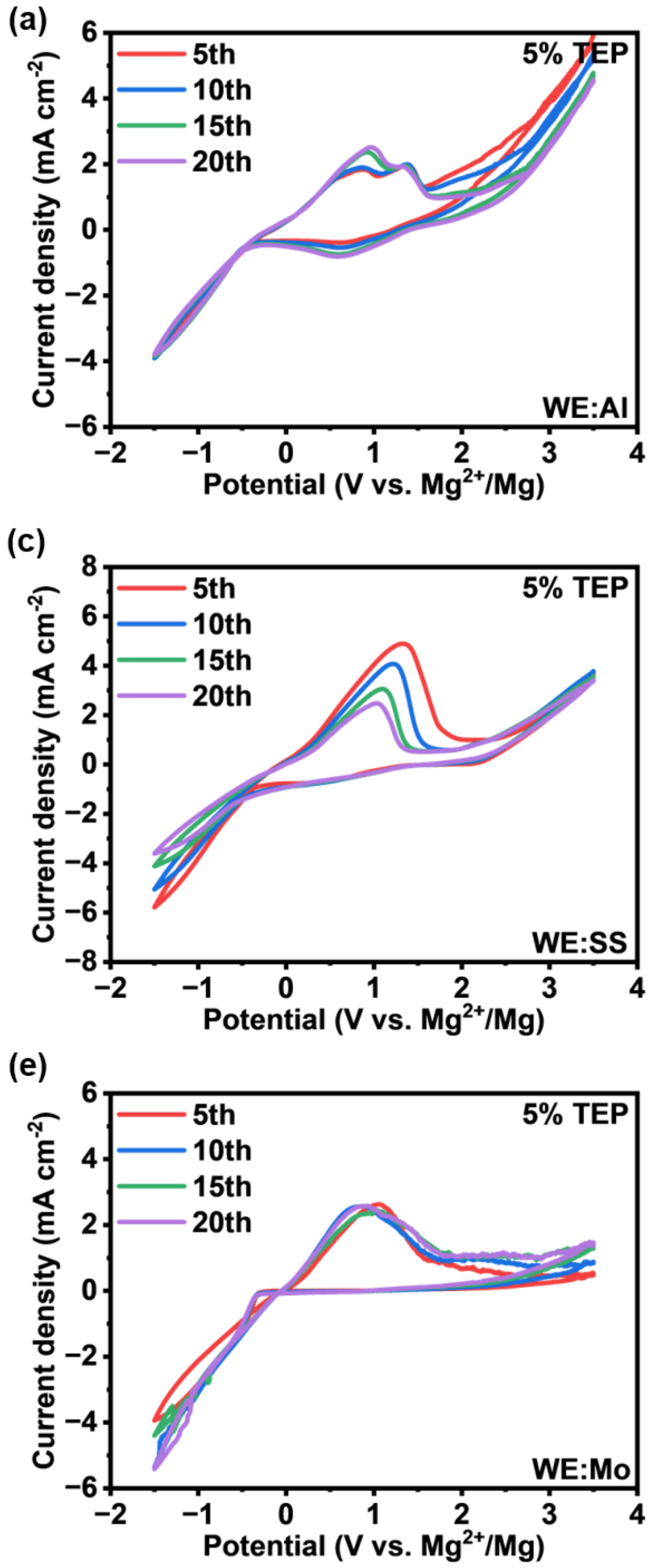

Figure S11. CV curves of Mg-based asymmetric cells in 0.3 M $Mg(OTf)_2$+0.2 M $MgCl_2$-DME electrolytes with 5% TEP using different working electrodes at a scan rate of 5 mV s^{-1} in a potential range of -1.5-3.5 V: (a) Al, (b) Cu, (c) SS, (d) Ti, (e) Mo.

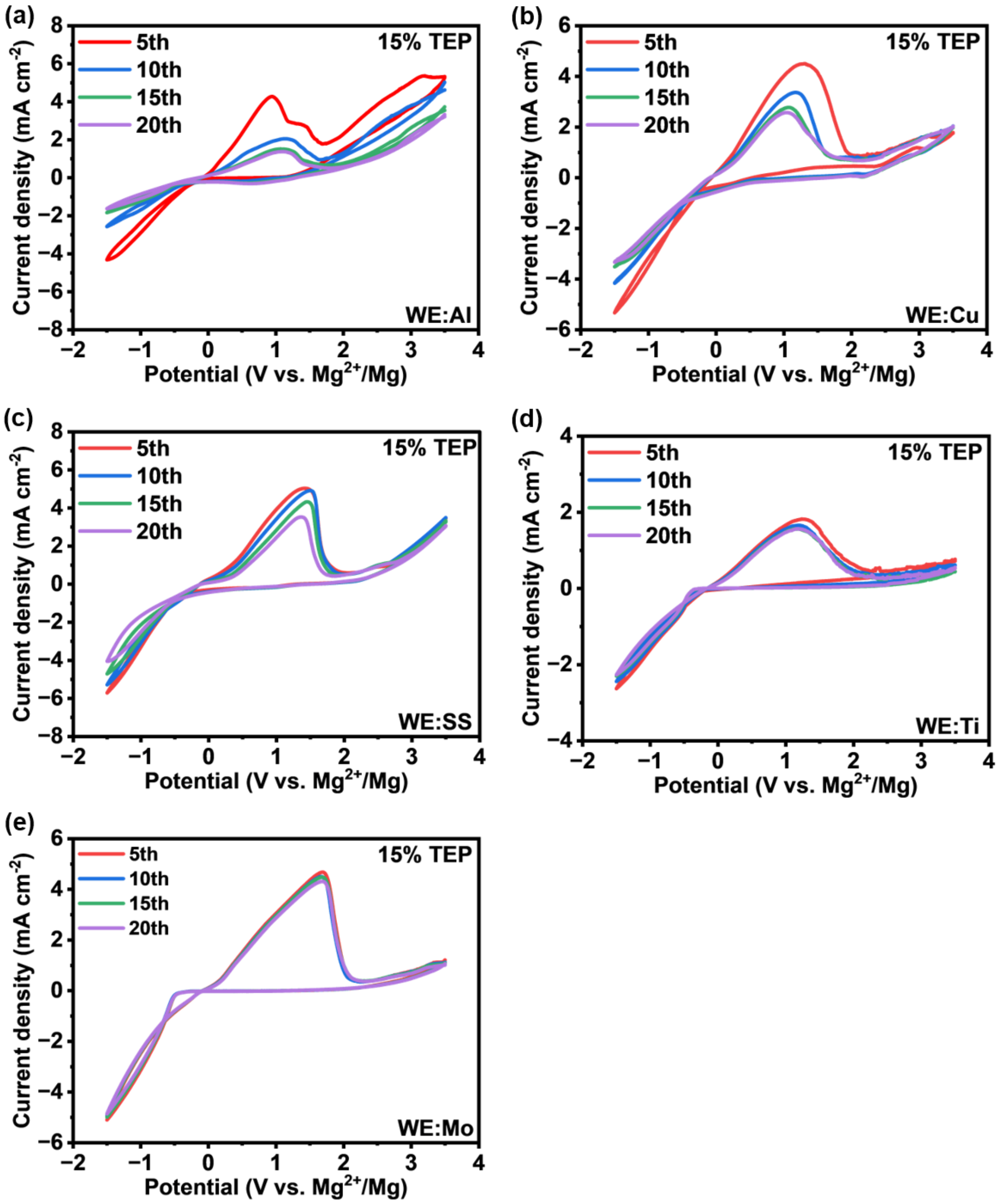

Figure S12. CV curves of Mg-based asymmetric cells in 0.3 M $Mg(OTf)_2$+0.2 M $MgCl_2$-DME electrolytes with 15% TEP using different working electrodes at a scan rate of 5 mV s^{-1} in a potential range of -1.5-3.5 V: (a) Al, (b) Cu, (c) SS, (d) Ti, (e) Mo.

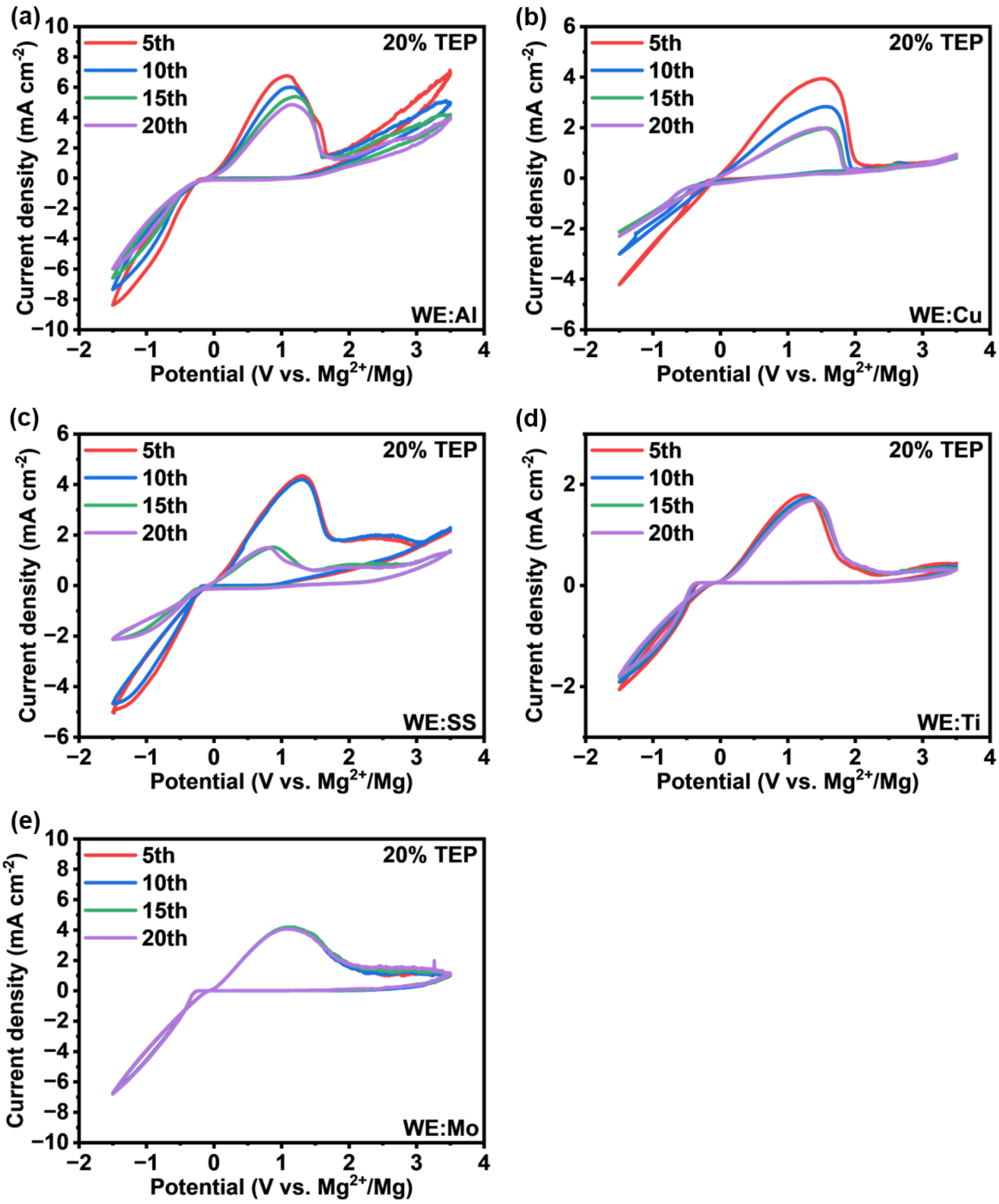

Figure S13. CV curves of Mg-based asymmetric cells in 0.3 M $Mg(OTf)_2$+0.2 M $MgCl_2$-DME electrolytes with 20% TEP using different working electrodes at a scan rate of 5 mV s^{-1} in a potential range of -1.5-3.5 V: (a) Al, (b) Cu, (c) SS, (d) Ti, (e) Mo.

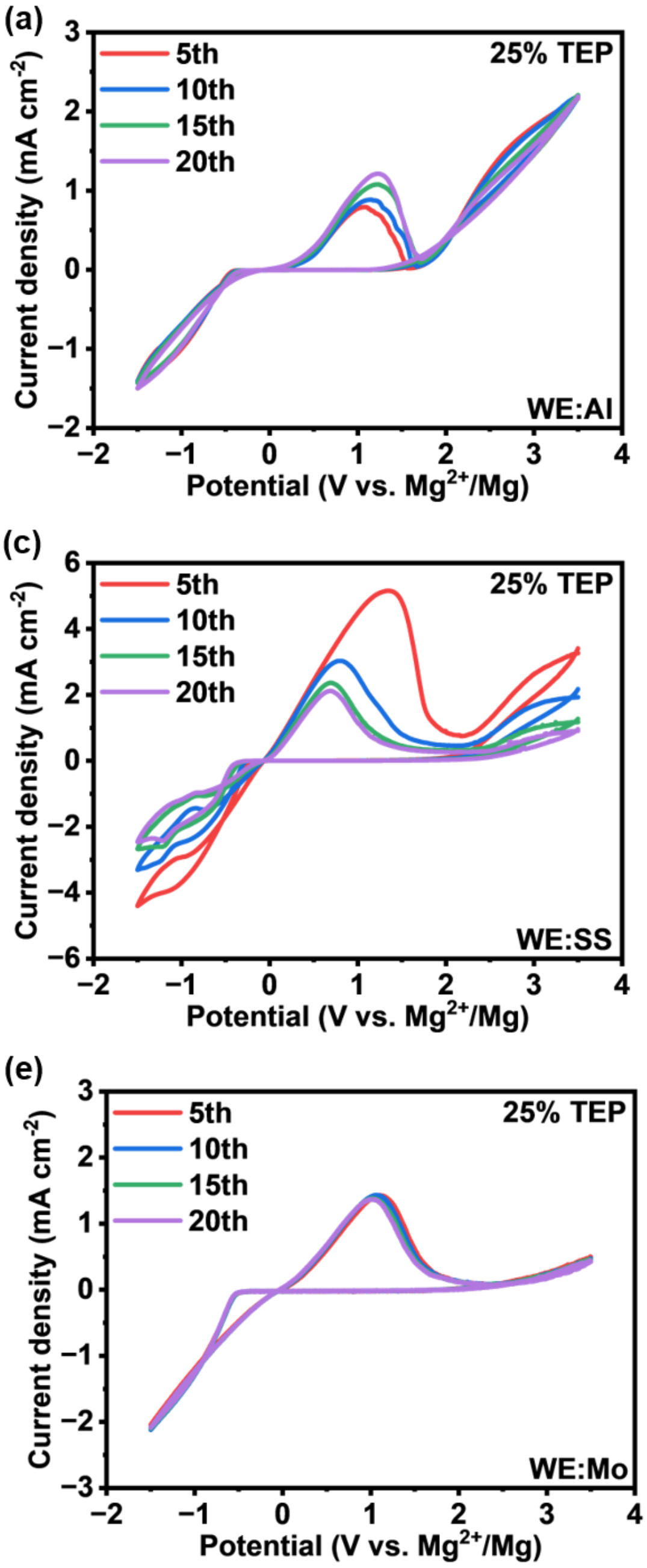

Figure S14. CV curves of Mg-based asymmetric cells in 0.3 M $Mg(OTf)_2$+0.2 M $MgCl_2$-DME electrolytes with 25% TEP using different working electrodes at a scan rate of 5 mV s^{-1} in a potential range of -1.5-3.5 V: (a) Al, (b) Cu, (c) SS, (d) Ti, (e) Mo.

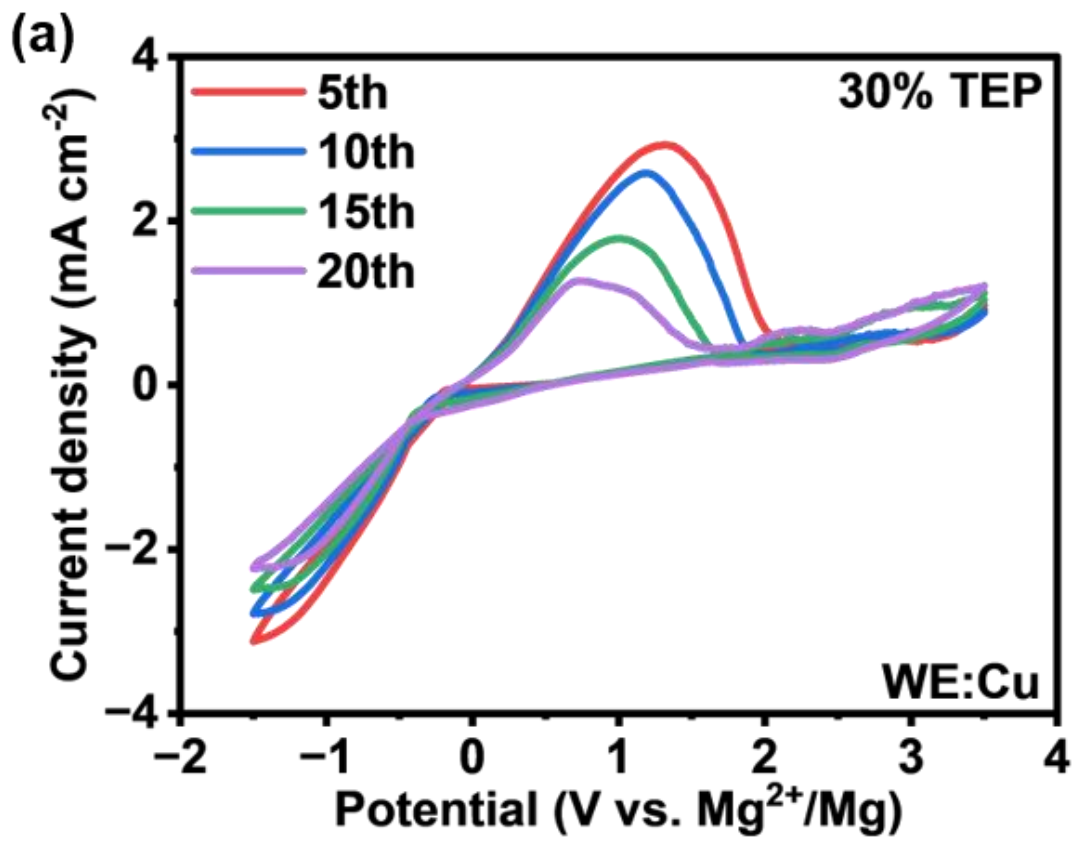

Figure S15. CV curves of Mg-based asymmetric cells in 0.3 M $Mg(OTf)_2$+0.2 M $MgCl_2$-DME electrolytes with 30% TEP using different working electrodes at a scan rate of 5 mV s^{-1} in a potential range of -1.5-3.5 V: (a) Cu, (b) Ti.

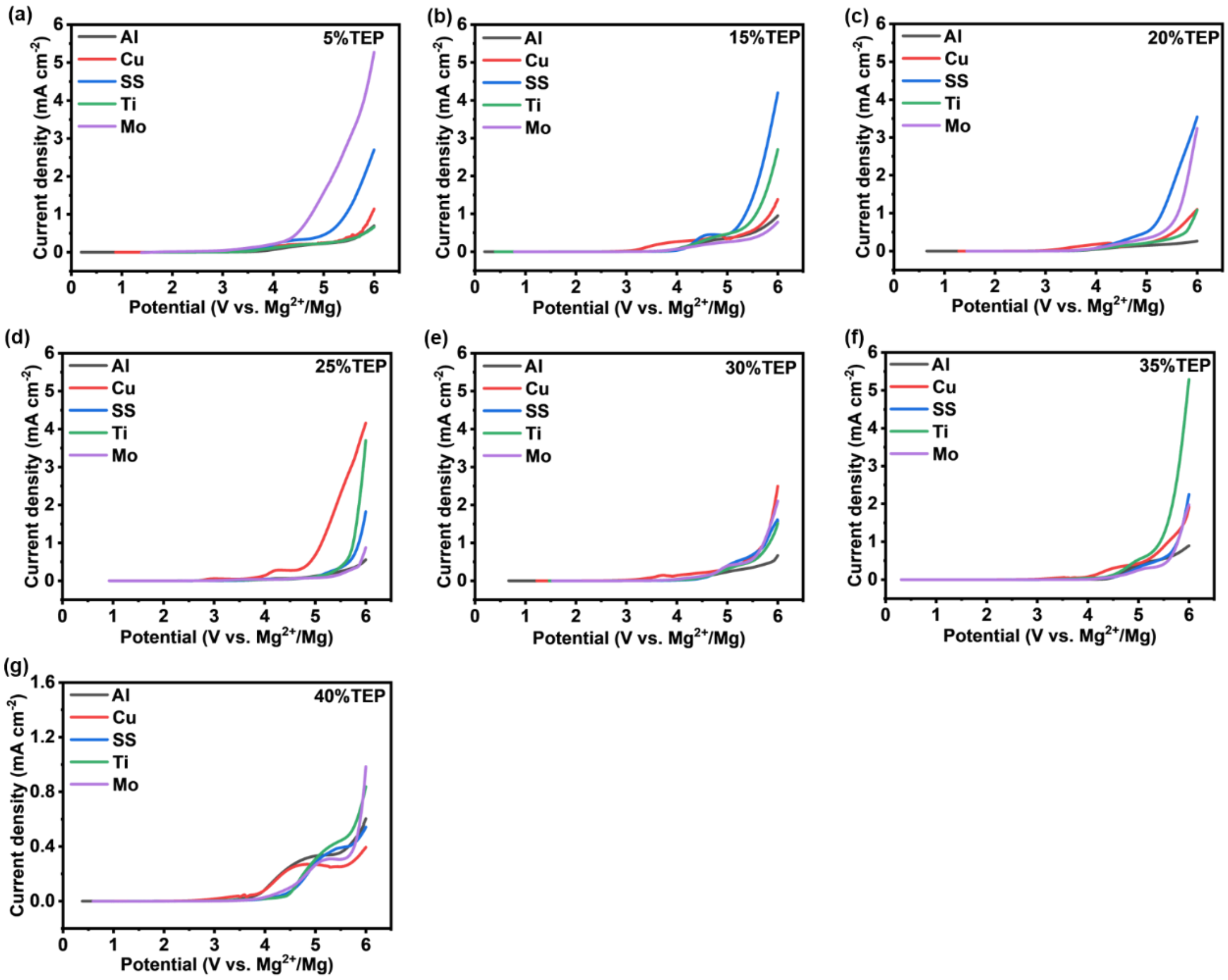

Figure S16. LSV curves of 0.3 M $Mg(OTf)_2$+0.2 M $MgCl_2$-DME electrolytes with varying TEP volume fractions using different working electrodes (Al, Cu, SS, Ti, Mo) at a scan rate of 5 mV s^{-1}: (a) 5% TEP, (b) 15% TEP, (c) 20% TEP, (d) 25% TEP, (e) 30% TEP, (f) 35% TEP, (g) 40% TEP.

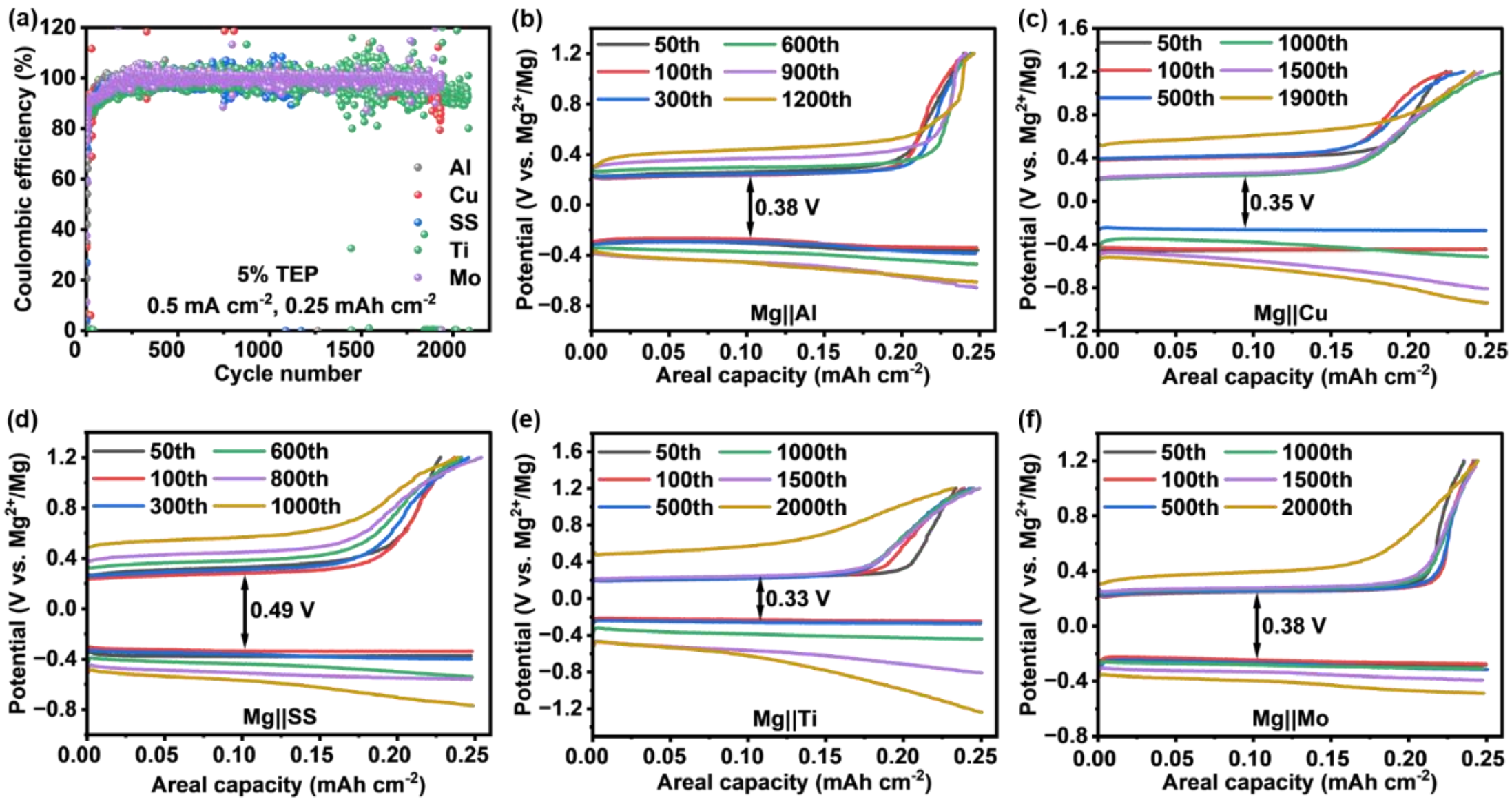

Figure S17. (a) CE and (b-f) voltage profiles of Mg-based asymmetric cells in 0.3 M $Mg(OTf)_2$+0.2 M $MgCl_2$-DME electrolytes with 5% TEP using different working electrodes (Al, Cu, SS, Ti, Mo) at 0.5 mA cm^{-2} and 0.25 mAh cm^{-2}.

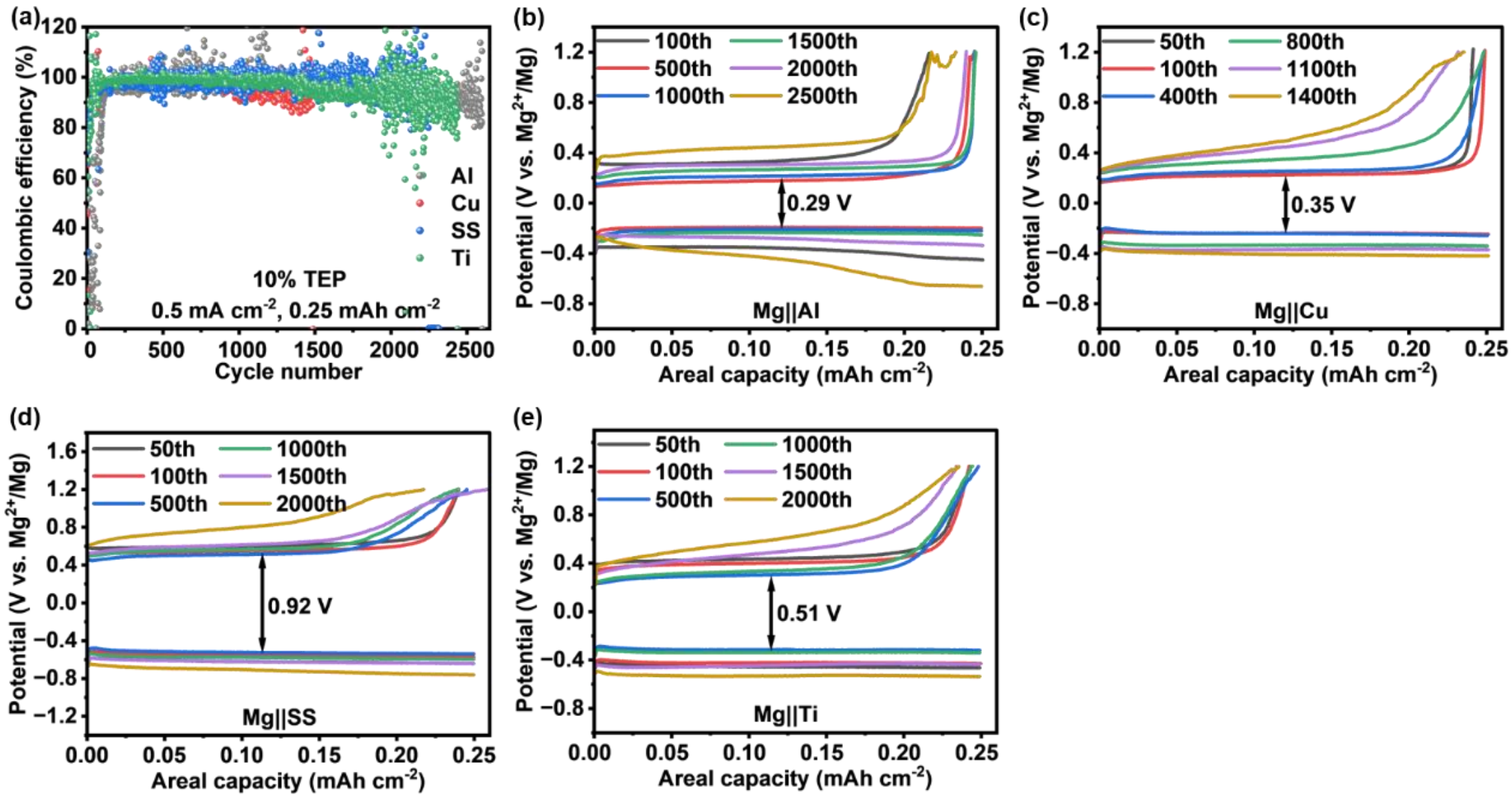

Figure S18. (a) CE and (b-e) voltage profiles of Mg-based asymmetric cells in 0.3 M $Mg(OTf)_2$+0.2 M $MgCl_2$-DME electrolytes with 10% TEP using different working electrodes (Al, Cu, SS, Ti, Mo) at 0.5 mA cm^{-2} and 0.25 mAh cm^{-2}.

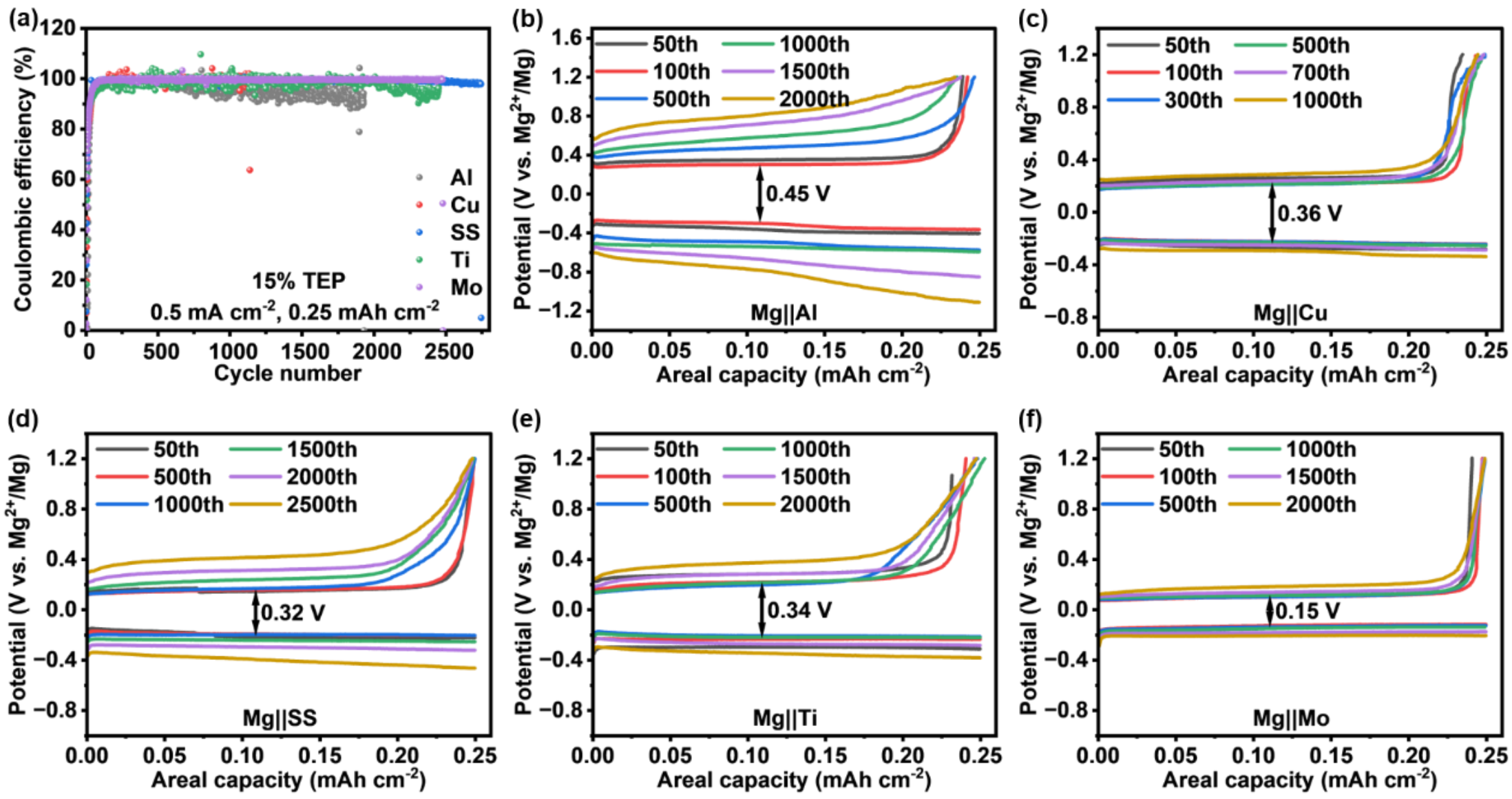

Figure S19. (a) CE and (b-e) voltage profiles of Mg-based asymmetric cells in 0.3 M $Mg(OTf)_2$+0.2 M $MgCl_2$-DME electrolytes with 15% TEP using different working electrodes (Al, Cu, SS, Ti, Mo) at 0.5 mA cm^{-2} and 0.25 mAh cm^{-2}.

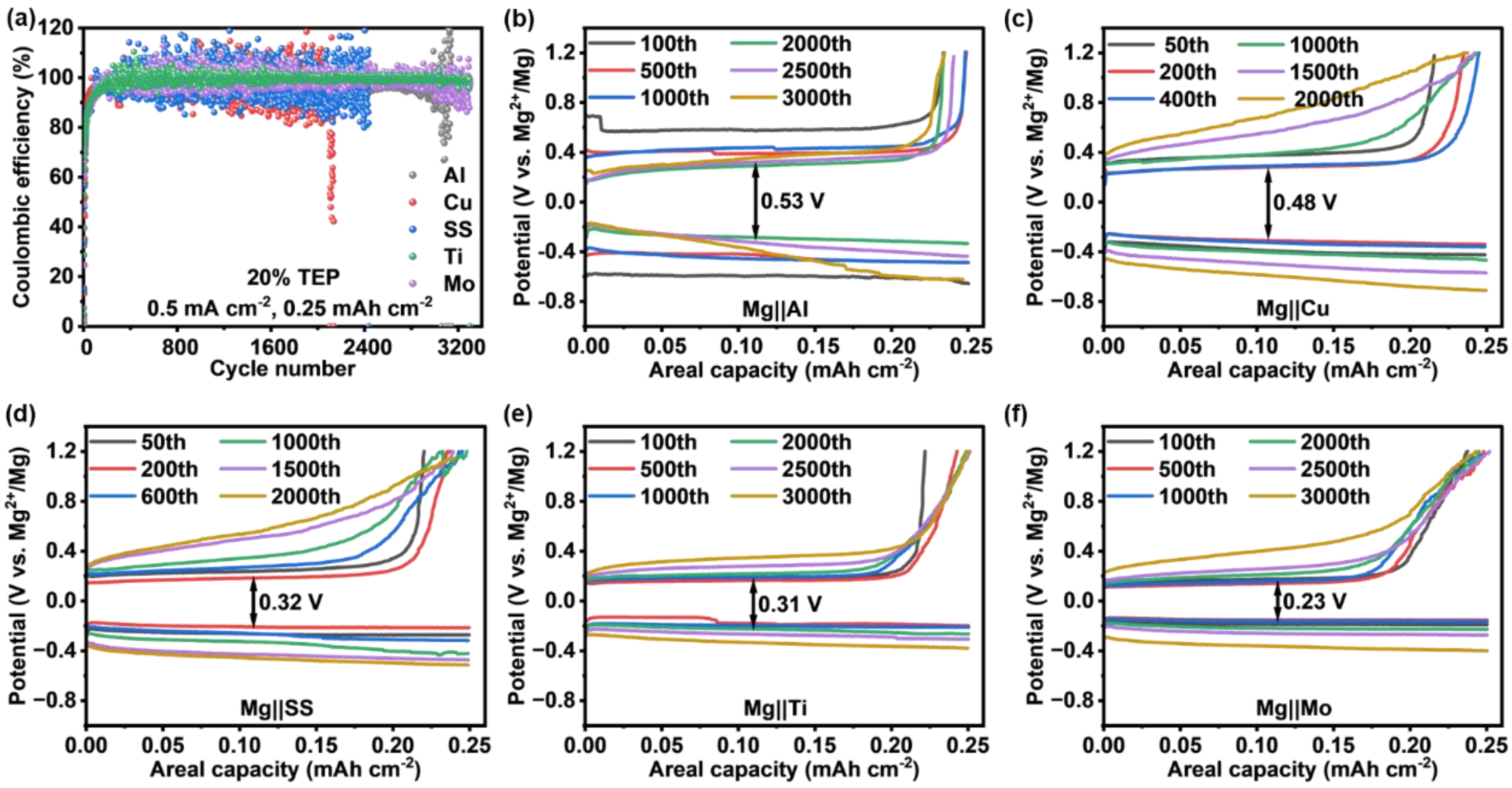

Figure S20. (a) CE and (b-e) voltage profiles of Mg-based asymmetric cells in 0.3 M $Mg(OTf)_2$+0.2 M $MgCl_2$-DME electrolytes with 20% TEP using different working electrodes (Al, Cu, SS, Ti, Mo) at 0.5 mA cm^{-2} and 0.25 mAh cm^{-2}.

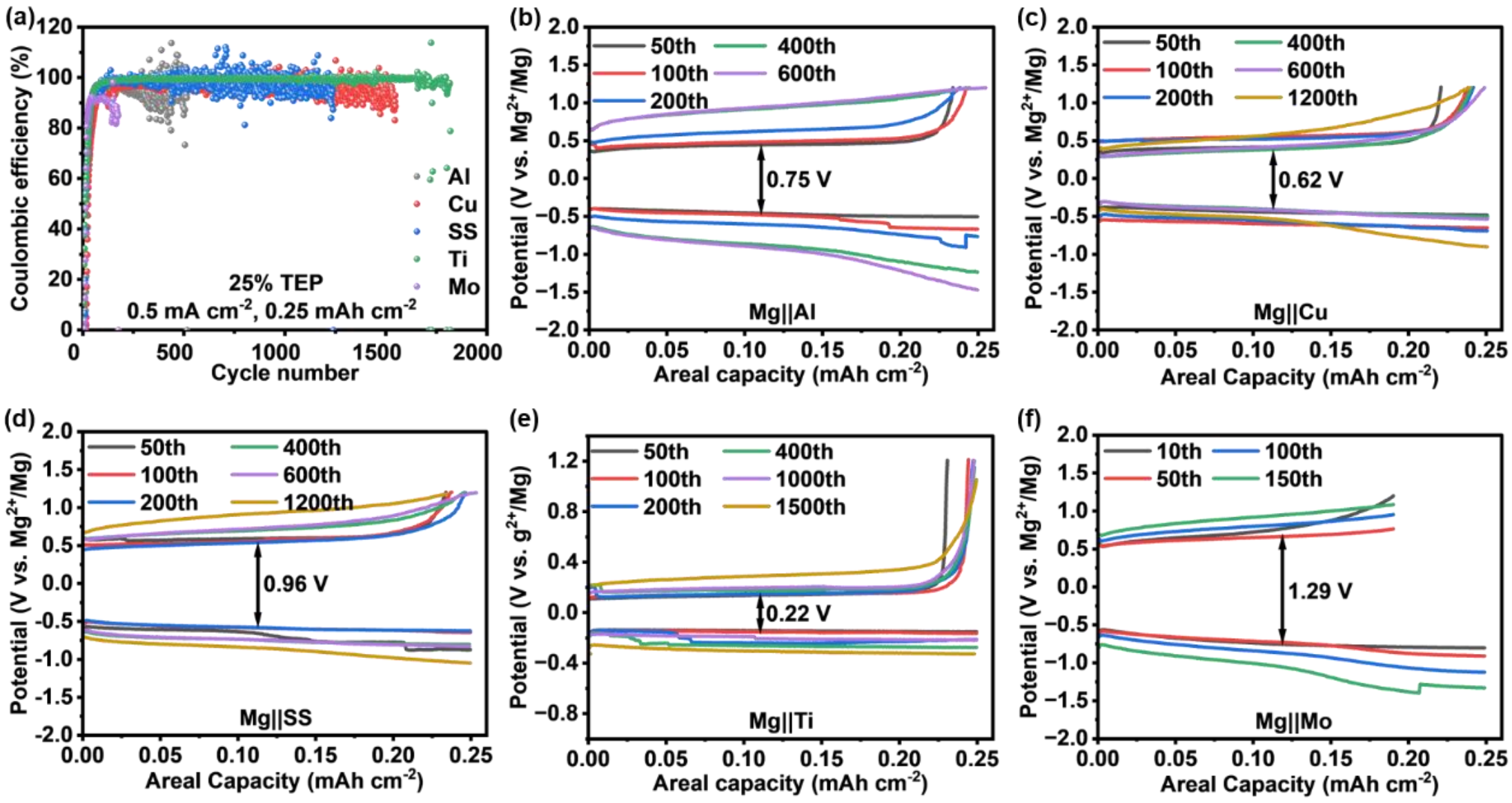

Figure S21. (a) CE and (b-e) voltage profiles of Mg-based asymmetric cells in 0.3 M $Mg(OTf)_2$+0.2 M $MgCl_2$-DME electrolytes with 25% TEP using different working electrodes (Al, Cu, SS, Ti, Mo) at 0.5 mA cm^{-2} and 0.25 mAh cm^{-2}.

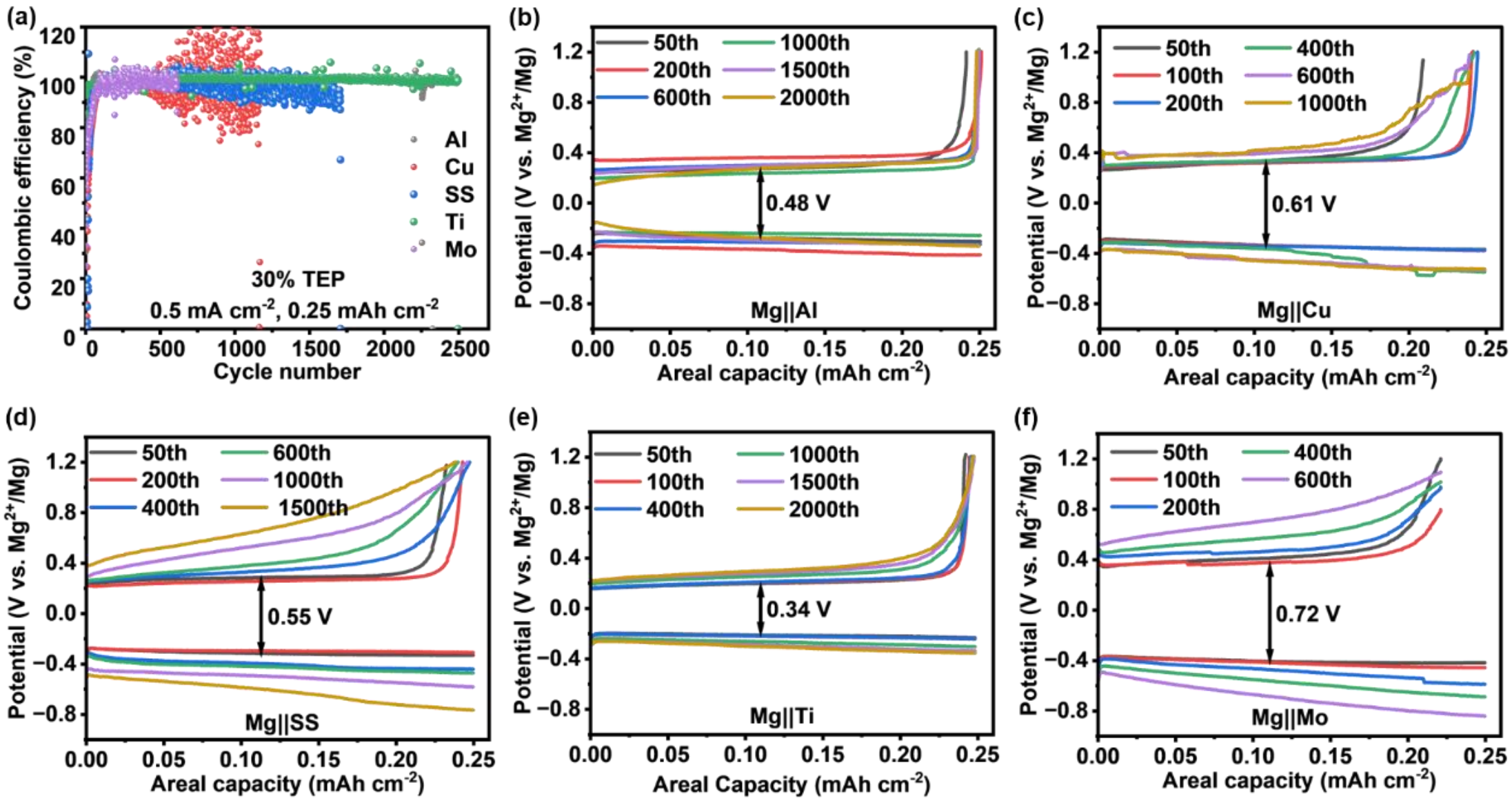

Figure S22. (a) CE and (b-e) voltage profiles of Mg-based asymmetric cells in 0.3 M $Mg(OTf)_2$+0.2 M $MgCl_2$-DME electrolytes with 30% TEP using different working electrodes (Al, Cu, SS, Ti, Mo) at 0.5 mA cm^{-2} and 0.25 mAh cm^{-2}.

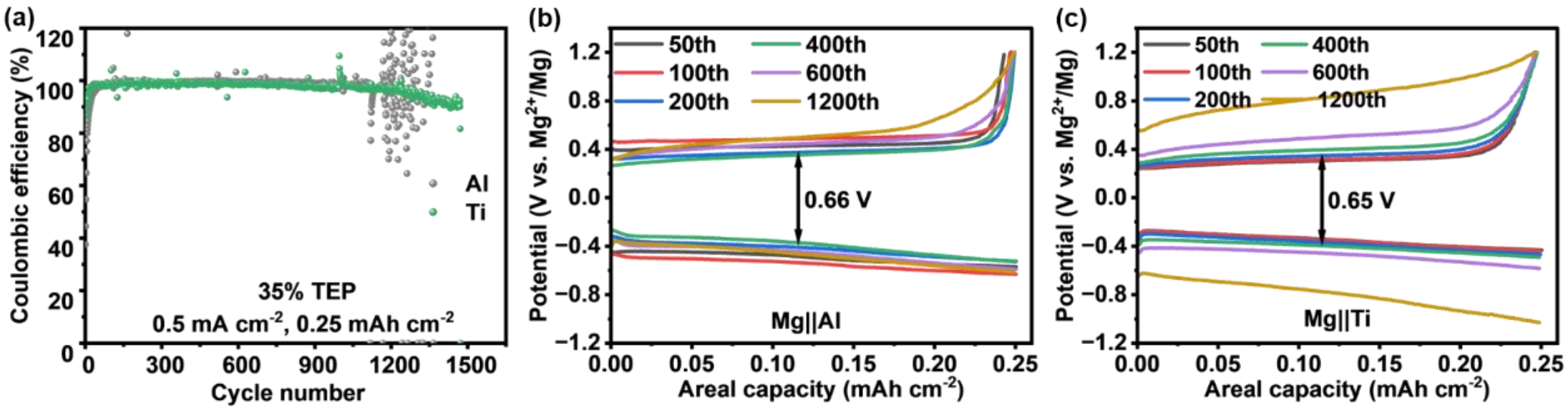

Figure S23. (a) CE and (b-e) voltage profiles of Mg-based asymmetric cells in 0.3 M $Mg(OTf)_2$+0.2 M $MgCl_2$-DME electrolytes with 35% TEP using different working electrodes (Al, Ti) at 0.5 mA cm^{-2} and 0.25 mAh cm^{-2}.

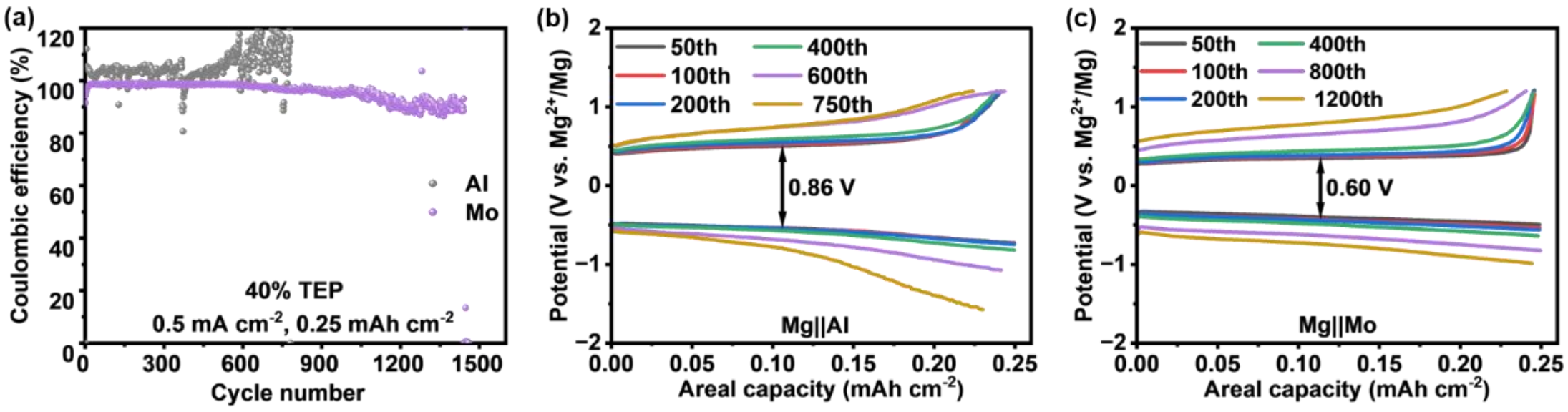

Figure S24. (a) CE and (b-e) voltage profiles of Mg-based asymmetric cells in 0.3 M $Mg(OTf)_2$+0.2 M $MgCl_2$-DME electrolytes with 40% TEP using different working electrodes (Al, Mo) at 0.5 mA cm^{-2} and 0.25 mAh cm^{-2}.

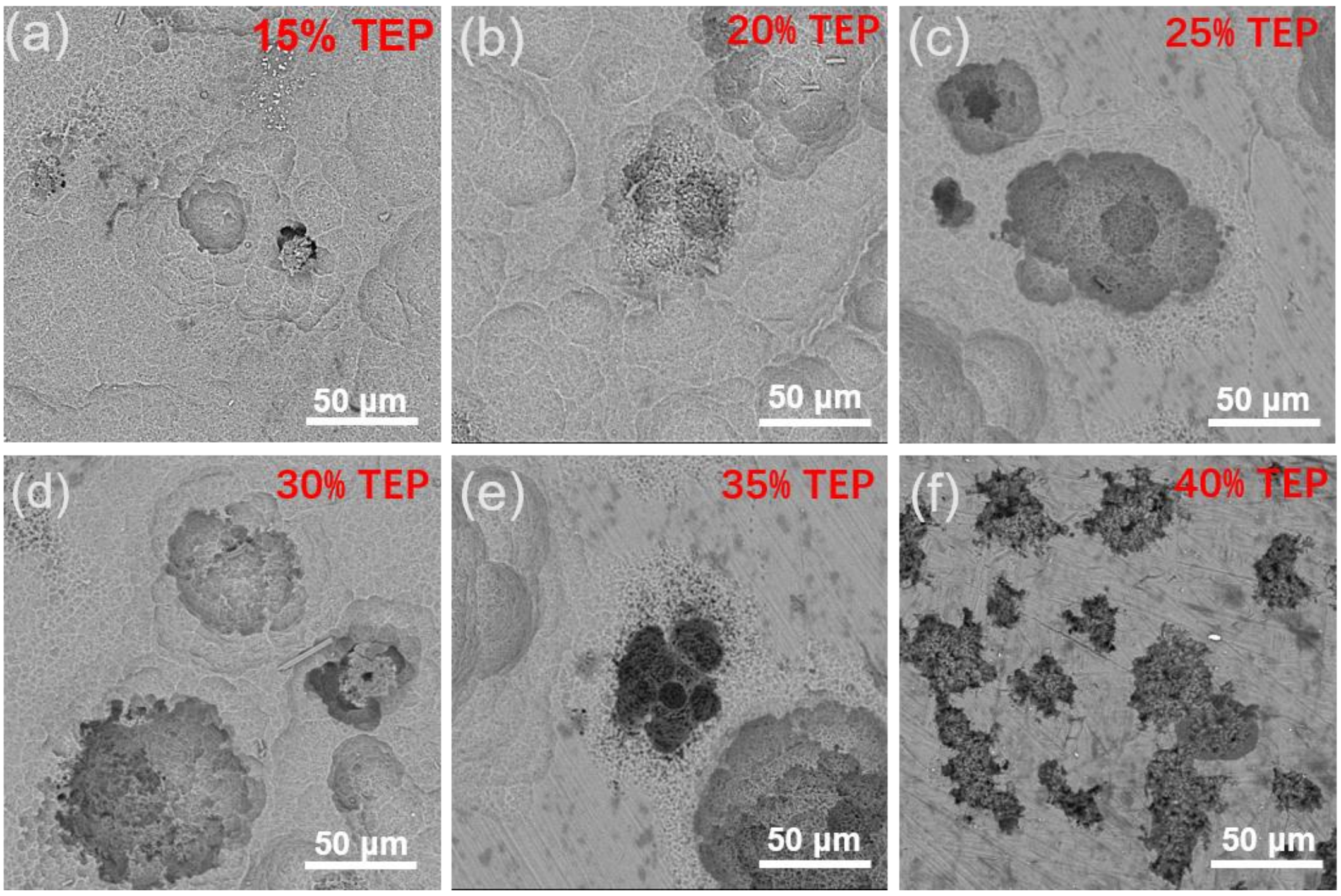

Figure S25. SEM images of Mg deposition from 0.3 M $Mg(OTf)_2$+0.2 M $MgCl_2$-DME electrolytes with varying TEP volume fractions in Mg||Mg symmetric cells after 100 cycles: (a) 15% TEP, (b) 20% TEP, (c) 25% TEP, (d) 30% TEP, (e) 35% TEP, (g) 40% TEP.

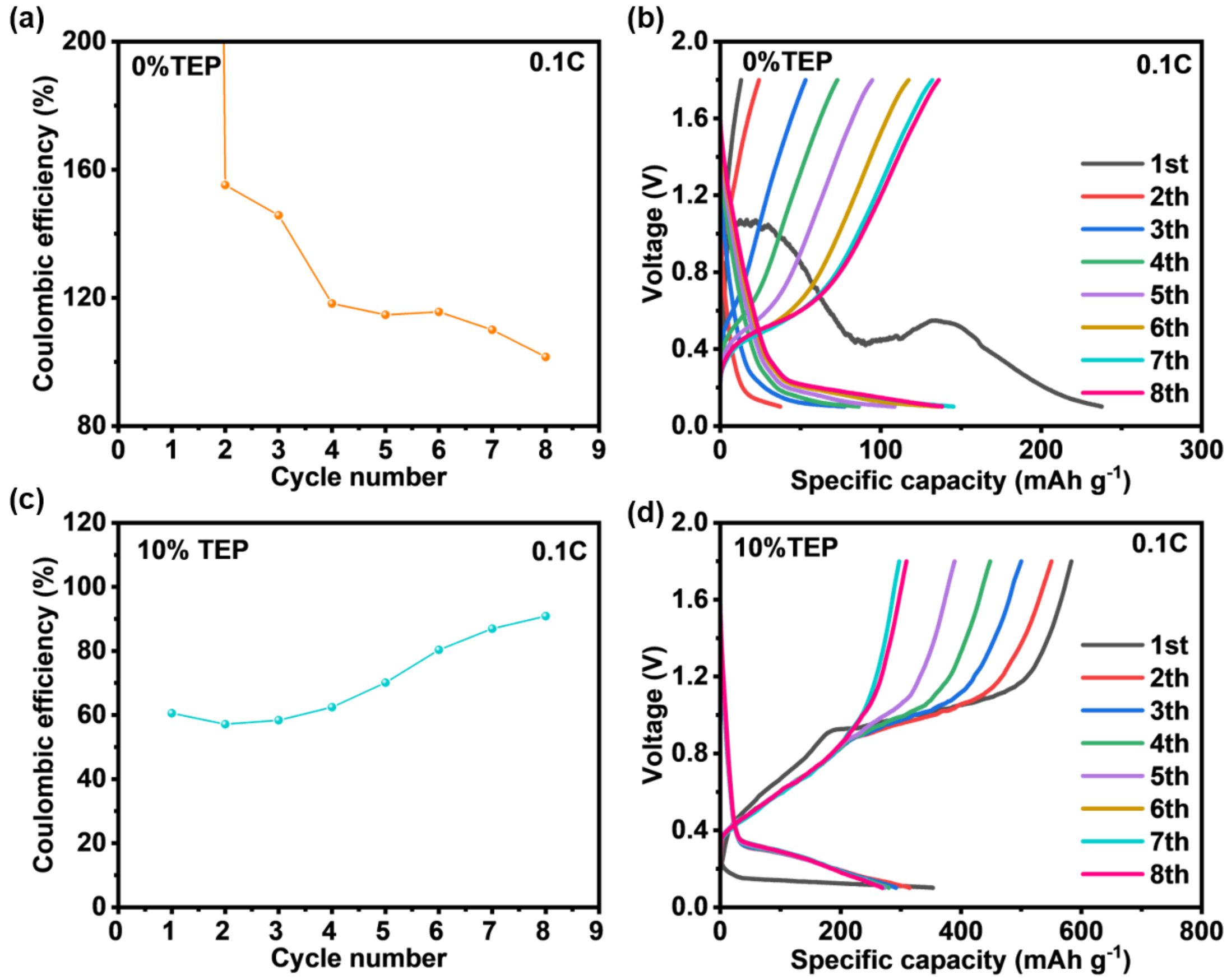

Figure S26. CEs and voltage profiles of the Mg||α-MnO_2 full cells during the initial eight cycles using (a-b) 0% TEP and (c-d) 10% TEP electrolytes at 0.1 C.

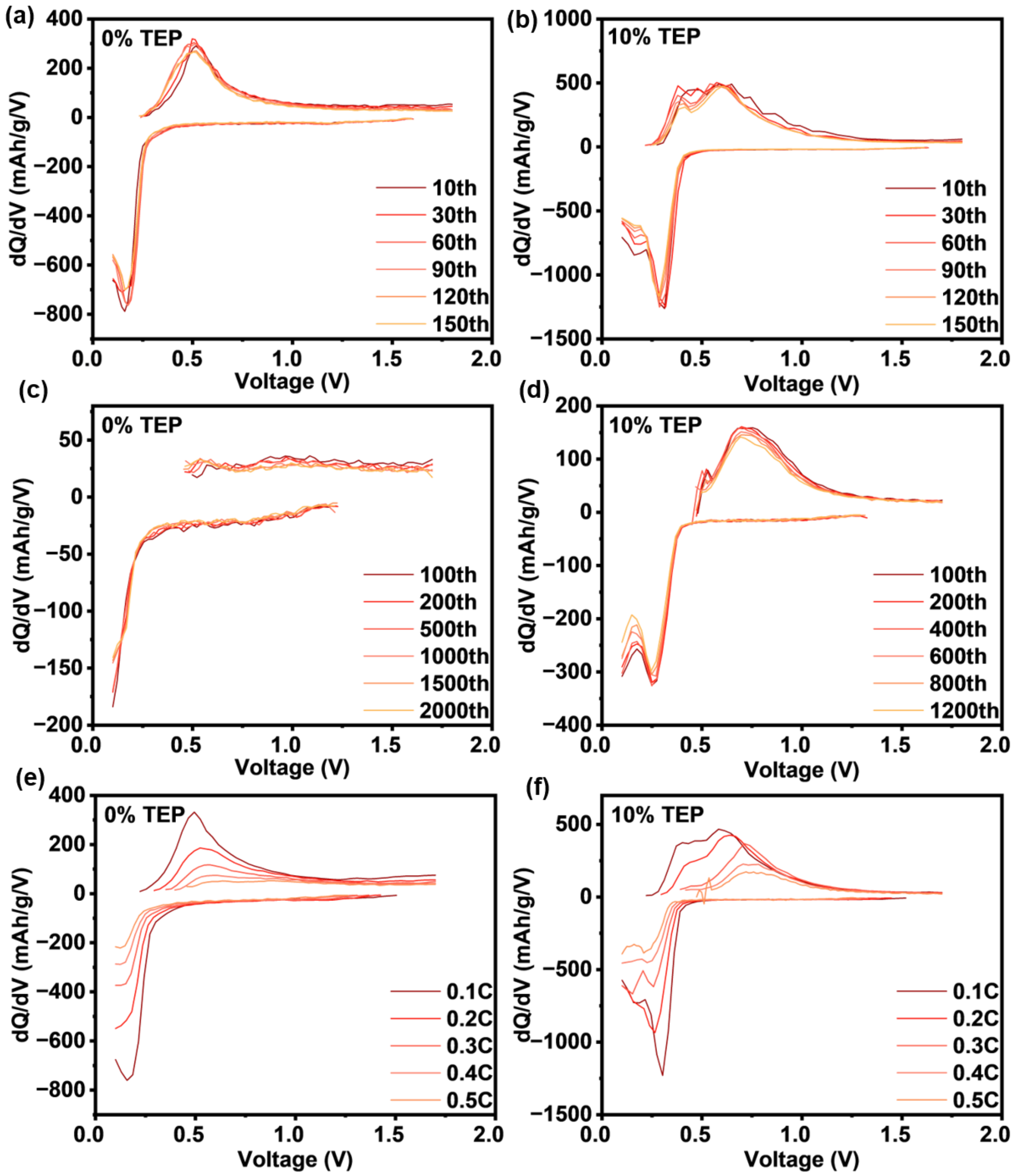

Figure S27. dQ/dV curves of long cycling in Mg||α-MnO_2 full cells with TEP-free and 10% TEP-modified electrolytes: (a) 0% TEP and (b) 10% TEP at 0.1C. (c) 0% TEP and (d) 10% TEP at 0.5C. dQ/dV curves of the rate capability in Mg||α-MnO_2 full cells: (e) 0% TEP and (f) 10% TEP from 0.1C to 0.5C.

Table S1. The fitted parameters of the EIS results cycled in Mg||Mg cells with 0% TEP and 10% TEP electrolytes.

**Mg||Mg Cells**	**0% TEP**			**10% TEP**		
**Cycle number**	R_S (Ω)	R_{SEI} (Ω)	R_{ct} (Ω)	R_S (Ω)	R_{SEI} (Ω)	R_{ct} (Ω)
**0**	56.69	281.6	2113000	45.12	56.51	715040
**50**	14.05	351.1	121980	111.2	237.7	25374
**100**	31.5	270.7	248930	32.2	136.4	33882

Table S2. Summary of proposed electroactive magnesium species in representative ether-based electrolytes reported in previous literatures and this work.

**Electrolytes**	**Reported/Proposed electroactive Mg species**	**Ref.**
0.25 M $AlCl_3$+0.5 M PhMgCl-THF	$[Mg_2Cl_3(THF)_6]^+$	1
0.25 M $AlCl_3$ + 0.25 M PhMgCl-THF	$[Mg_2Cl_3(THF)_6]^+$	1
$MgCl_2$:$AlCl_3$ (2:1)-THF	$[MgCl^+\cdot 3THF]$	2
$MgCl_2$:$AlCl_3$ (2:1)-THF	$[Mg_2Cl_3(THF)_6]^+$	3
$MgCl_2$:$AlCl_3$ (1:1)-THF	$[Mg(THF)_6]^{2+}$	4
$MgCl_2$:$AlCl_3$ (2:1)-THF	$[(\mu\text{-}Cl)_3Mg_2(THF)_6]^+$	4
0.4 M $MgCl_2$ + 0.4 M $AlEtCl_2$-DME	$[Mg_2(\mu\text{-}Cl)_2(DME)_4]^{2+}$	5
0.4 M $MgCl_2$ + 0.4 M $AlCl_3$-DME	$[Mg_2(\mu\text{-}Cl)_2(DME)_4]^{2+}$	5
0.4 M $MgCl_2$ + 0.4 M $Mg(TFSI)_2$-DME	$[Mg_2(\mu\text{-}Cl)_2(DME)_4]^{2+}$	5
0.5 M $MgCl_2$ + 0.5 M $Mg(TFSI)_2$-DME	$[Mg_2(\mu\text{-}Cl)_2(DME)_4]^{2+}$	6
1 M $MgCl_2$ + 0.5 M $Mg(TFSI)_2$-DME	$[Mg_3(\mu\text{-}Cl)_4(DME)_5]^{2+}$	6
0.2 M $MgCl_2$+ 0.3 M $Mg(OTf)_2$-DME	$Mg(OTf)_2(DME)_2$, $[Mg(DME)_3]^{2+}$	7
0.2 M $MgCl_2$ + 0.3 M $Mg(OTf)_2$-DME+TEP	$[Mg(DME)_x(TEP)_y]^{2+}$	This work

References

(1) Pour, N.; Gofer, Y.; Major, D. T.; Aurbach, D. Structural Analysis of Electrolyte Solutions for Rechargeable Mg Batteries by Stereoscopic Means and DFT Calculations. *J. Am. Chem. Soc.* **2011**, *133* (16), 6270–6278. https://doi.org/10.1021/ja1098512.

(2) Canepa, P.; Jayaraman, S.; Cheng, L.; Rajput, N. N.; Richards, W. D.; Gautam, G. S.; Curtiss, L. A.; Persson, K. A.; Ceder, G. Elucidating the Structure of the Magnesium Aluminum Chloride Complex Electrolyte for Magnesium-Ion Batteries. *Energy Environ. Sci.* **2015**, *8* (12), 3718–3730. https://doi.org/10.1039/C5EE02340H.

(3) See, K. A.; Liu, Y.-M.; Ha, Y.; Barile, C. J.; Gewirth, A. A. Effect of Concentration on the Electrochemistry and Speciation of the Magnesium Aluminum Chloride Complex Electrolyte Solution. *ACS Appl. Mater. Interfaces* **2017**, *9* (41), 35729–35739. https://doi.org/10.1021/acsami.7b08088.

(4) Chaffin, V. O.; Alves, W. A. Composition Dependence on the Spectral Behavior of Magnesium Aluminum Chloride Complex Electrolytes. *J. Mol. Liq.* **2020**, *315*, 113722.

https://doi.org/10.1016/j.molliq.2020.113722.
(5) Cheng, Y.; Stolley, R. M.; Han, K. S.; Shao, Y.; Arey, B. W.; Washton, N. M.; Mueller, K. T.; Helm, M. L.; Sprenkle, V. L.; Liu, J.; Li, G. Highly Active Electrolytes for Rechargeable Mg Batteries Based on a $[Mg_2 (\mu\text{-}Cl)_2]^{2+}$ Cation Complex in Dimethoxyethane. *Phys. Chem. Chem. Phys.* **2015**, *17* (20), 13307–13314. https://doi.org/10.1039/C5CP00859J.
(6) Salama, M.; Shterenberg, I.; J.W. Shimon, L.; Keinan-Adamsky, K.; Afri, M.; Gofer, Y.; Aurbach, D. Structural Analysis of Magnesium Chloride Complexes in Dimethoxyethane Solutions in the Context of Mg Batteries Research. *J. Phys. Chem. C* **2017**, *121* (45), 24909–24918. https://doi.org/10.1021/acs.jpcc.7b05452.
(7) Nguyen, D.-T.; Eng, A. Y. S.; Ng, M.-F.; Kumar, V.; Sofer, Z.; Handoko, A. D.; Subramanian, G. S.; Seh, Z. W. A High-Performance Magnesium Triflate-Based Electrolyte for Rechargeable Magnesium Batteries. *Cell Rep. Phys. Sci.* **2020**, *1* (12), 100265. https://doi.org/10.1016/j.xcrp.2020.100265.
